# Downstream Warming and Headwater Acidity May Diminish Coldwater Habitat in Southern Appalachian Mountain Streams

**DOI:** 10.1371/journal.pone.0134757

**Published:** 2015-08-06

**Authors:** T. C. McDonnell, M. R. Sloat, T. J. Sullivan, C. A. Dolloff, P. F. Hessburg, N. A. Povak, W. A Jackson, C. Sams

**Affiliations:** 1 E&S Environmental Chemistry, Inc., Corvallis, Oregon, United States of America; 2 Department of Forest Engineering, Resources and Management, Oregon State University, Corvallis, Oregon, United States of America; 3 USDA Forest Service, Southern Research Station, Blacksburg, Virginia, United States of America; 4 USDA Forest Service, Pacific Northwest Research Station, Forestry Sciences Laboratory, Wenatchee, Washington, United States of America; 5 USDA Forest Service, Asheville, North Carolina, United States of America; 6 USDA Forest Service, Regions 8 and 9 Air Quality Program, Atlanta, Georgia, United States of America; University of Yamanashi, JAPAN

## Abstract

Stream-dwelling species in the U.S. southern Appalachian Mountains region are particularly vulnerable to climate change and acidification. The objectives of this study were to quantify the spatial extent of contemporary suitable habitat for acid- and thermally sensitive aquatic species and to forecast future habitat loss resulting from expected temperature increases on national forest lands in the southern Appalachian Mountain region. The goal of this study was to help watershed managers identify and assess stream reaches that are potentially vulnerable to warming, acidification, or both. To our knowledge, these results represent the first regional assessment of aquatic habitat suitability with respect to the combined effects of stream water temperature and acid-base status in the United States. Statistical models were developed to predict July mean daily maximum water temperatures and air-water temperature relations to determine potential changes in future stream water temperatures. The length of stream considered suitable habitat for acid- and thermally sensitive species, based on temperature and acid neutralizing capacity thresholds of 20°C and 50 μeq/L, was variable throughout the national forests considered. Stream length displaying temperature above 20°C was generally more than five times greater than the length predicted to have acid neutralizing capacity below 50 μeq/L. It was uncommon for these two stressors to occur within the same stream segment. Results suggested that species’ distributional shifts to colder, higher elevation habitats under a warming climate can be constrained by acidification of headwater streams. The approach used in this study can be applied to evaluate climate change impacts to stream water resources in other regions.

## Introduction

Climate change presents a central challenge to the maintenance of native aquatic biodiversity [1]. Stream-dwelling species are particularly vulnerable to climate change because most are ectothermic and exist only within narrow ranges of temperature and water chemistry, and their movements are constrained to easily fragmented linear networks [2, 3]. A growing number of studies demonstrate that riverine ecosystems are warming outside the recent historical range [4, 5], and that many coldwater species within stream networks are undergoing range contractions or shifts in distribution to higher elevation [2, 6–8]. Consequently, the need to understand how regional climate projections will likely influence local (e.g. stream reach) abiotic conditions and associated biota has intensified. Managers must acquire new tools to assess the vulnerability of stream reaches and to identify potential refugia for aquatic organisms (see [3, 9, 10]).

In addition to water temperature, other stream characteristics may constrain species occurrence and act with temperature to influence the spatial distribution of habitat and potential thermal refugia. For example, in the U.S. southern Appalachian Mountains (SAM) region, stream acidification is an important limiting influence on aquatic habitat suitability [11, 12]. Many SAM watersheds are vulnerable to stream acidification caused by atmospherically deposited acidifying compounds containing sulfur (S) and nitrogen (N) because some soils within the region are inherently low in base cation (Ca^2+^, Mg^2+^, K^+^, and Na^+^) buffering capacity as a result of low mineral weathering rates [13–15]. Weathered base cations dissolve in soil water and move into streams where they can neutralize acidity associated with strong mineral acid anions (nitrate [NO_3_^-^]) and sulfate ([SO_4_^2-^]). This acid-neutralizing capacity (ANC) is a key measure of stream water acid-base chemistry that is associated with reduced fitness and richness of aquatic biota in the SAM region [12] and elsewhere [16]. Low background (pre-industrial) stream water base cation concentrations limit stream water ANC in some SAM watersheds. This situation has been exacerbated by decades of atmospheric S and N deposition, which has caused deleterious effects on fish and stream macroinvertebrate communities [12, 17].

Although atmospheric contributions of S and N have declined over the past two to three decades throughout the eastern United States in response to the Clean Air Act and other regulations [18], streams with inherently low ANC will likely remain vulnerable well into the future because of chronic depletion of the soil base cation pool and diminished capacity for soil adsorption of continued inputs of anthropogenic S [17, 19]. Stream acidification is and will remain a central concern for managers of SAM watersheds, and this concern is magnified by the potential threat of additional habitat loss from climate-induced stream warming [20, 21].

The combined effect of thermal habitat loss and stream acidification presents a conundrum for watershed managers tasked with identifying suitable habitats for coldwater species (e.g., brook trout [*Salvelinus fontinalis*]) [10, 21]. In general, low-order, higher-elevation streams are most susceptible to acidification; in the absence of adequate buffering capacity, otherwise suitable habitat is rendered uninhabitable in the headwaters of susceptible streams [22, 23]. In contrast, thermal habitat for coldwater species becomes less suitable (i.e., warmer) as elevation decreases. We therefore hypothesized that i) these two stressors may exhibit minimal spatial overlap within stream networks of the SAM under contemporary conditions, and ii) climate induced stream warming will further constrain suitable habitat for acid-sensitive coldwater aquatic species to mid-elevation portions of stream networks, increasing the likelihood of both local and regional species extirpation.

**Fig 1:**
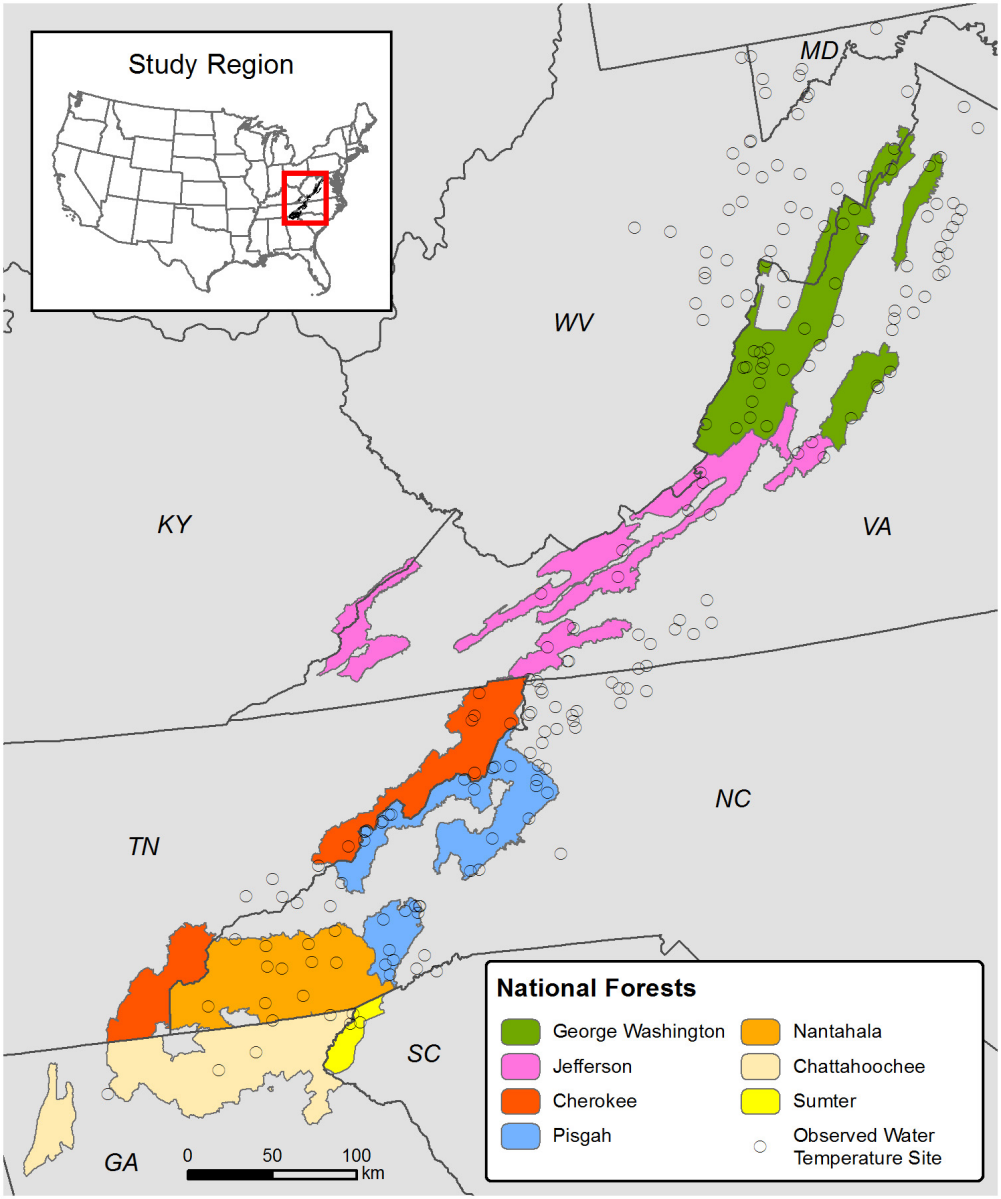
Map showing national forest proclamation boundaries within the study region and locations of observed stream water temperature sites.

The primary objectives of our study were to test our hypothesis by developing first approximations of the spatial extent of contemporary suitable habitat for acid- and thermally sensitive aquatic species and to forecast habitat loss resulting from expected future air temperature increases throughout the SAM region. We analyzed the spatial distribution of stream water ANC in relation to contemporary and projected future stream temperatures. Our use of the phrase “stream temperature” corresponds with the water temperature of the stream and does not refer to temperature of the stream bed sediment or other stream-related feature. This study was focused on streams draining seven U.S. national forests (NFs) within the region (Fig 1). Our spatially explicit assessment will help watershed managers assess the need for potential intervention (liming, riparian afforestation, native fish reintroduction) in stream reaches that are potentially vulnerable to warming, acidification, or both.

## Methods

### Study sites

The study area included a combination of public and private land within the proclamation boundaries that encompass forestlands managed by the USDA Forest Service (USFS; Fig 1). The study area lies within the oak-hickory forest region [24] and contains portions of the Ridge and Valley, Blue Ridge, and a small portion of the Piedmont ecoregions. Forest types are diverse, and include forest communities dominated by oaks and tulip poplar, pines, and northern hardwoods and spruce-fir at the highest elevations.

The stream network we used was generated from hydrologically-conditioned digital elevation model (DEM) derivatives from the NHDPlus database [25]. This allowed us to develop a custom grid-based stream network at an appropriate analysis scale and facilitated calculation of explanatory variables [14]. We designated a minimum watershed size for headwater streams as 0.5 km^2^; downstream watersheds were determined according to stream junctions. A minimum watershed size is required for defining headwater stream generation. Topographically derived streams corresponded reasonably well with those depicted in the high-resolution National Hydrography Dataset [26], and the designation process resulted in a total of 38,763 watersheds with a total area of 35,783 km^2^. Incremental drainage area, which is the additional drainage area included when moving downstream from one stream segment to the next, averaged 0.9 km^2^ in size (median = 0.7; interquartile range = 0.4 to 1.2 km^2^). Individual stream reaches associated with these incremental drainage areas provided the basis for extrapolation of modeling results to the full stream network.

### Vulnerability assessment approach

**Fig 2:**
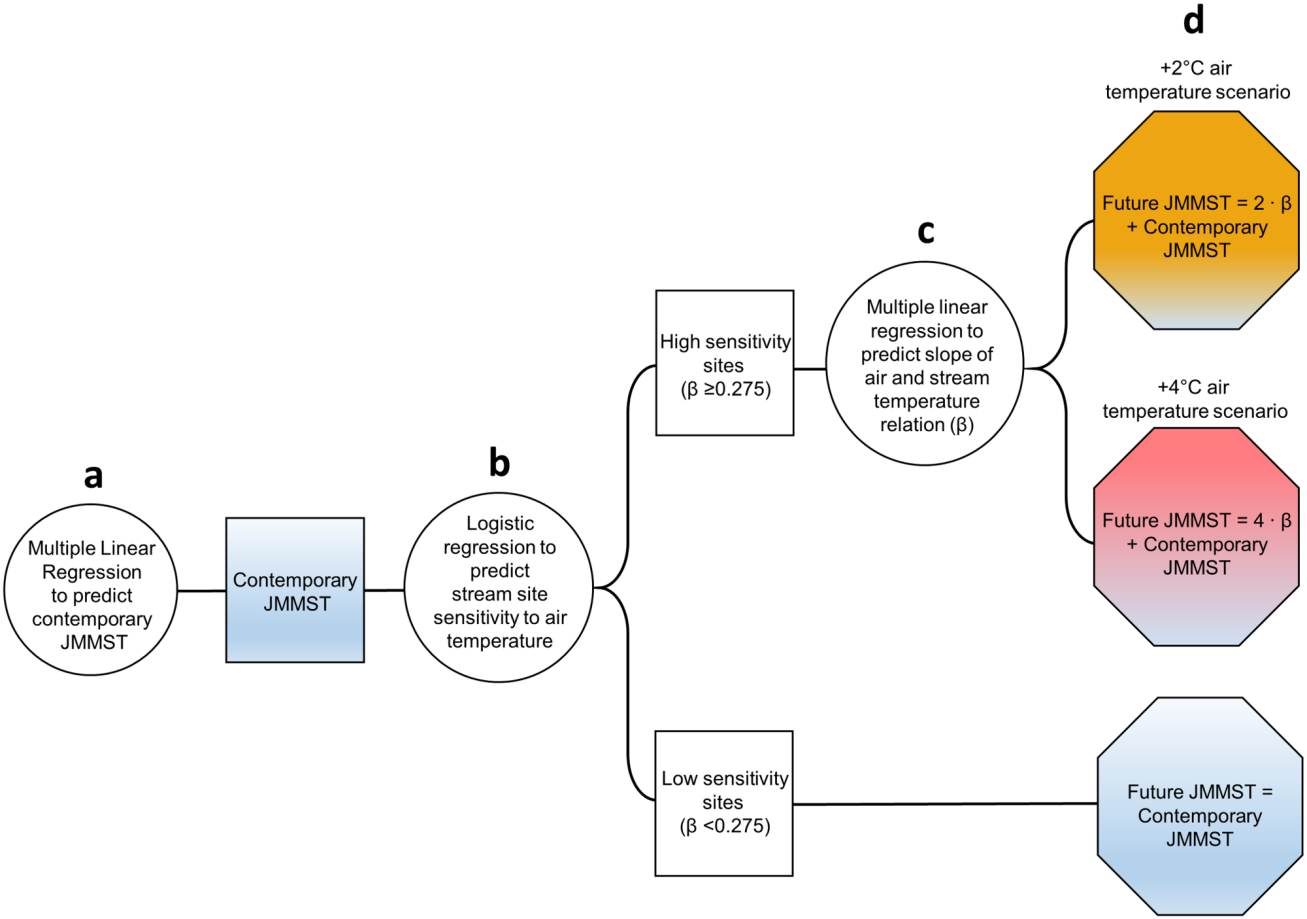
Conceptual diagram of the modeling framework used to develop contemporary and future estimates of July mean daily maximum stream water temperature (JMMST) for the study region. The diagram shows how the three regression modeling steps (a, b, c) were used to develop the final set of model results (d).

Our general approach for assessing the combined effects of stream warming and acidification on aquatic habitat in the SAM involved the following steps:

1. Develop a correlative model to predict July mean daily maximum stream water temperature (JMMST) under contemporary climate conditions (Fig 2a).
2. Model future JMMST associated with July atmospheric warming of 2 and 4°C by (*i*) developing a predictive threshold model to identify stream reaches with high sensitivity to warming air temperatures, which were defined by stream and air temperature correlations (Fig 2b); and (*ii*) developing a predictive model for high-sensitivity stream sites to derive continuous estimates of the strength of stream water and air temperature correlations (Fig 2c).
3. Refine previously published regional stream ANC estimates [23] by correcting for model bias and re-projecting a continuous ANC surface across the study region.
4. Map refined ANC and JMMST estimates to quantify potential loss of stream habitat for acid-sensitive coldwater species.

### Stream temperature models

#### Contemporary stream temperature

To characterize a contemporary period of annual maximum stream water temperatures, we developed multiple linear regression models to predict JMMST from climate and landscape characteristics (Fig 2a). Models were parameterized with stream temperature data measured at 231 sites within the SAM that had minimally disturbed hydrology (e.g., no major upstream dams or water withdrawals). These data were collected in 2012 by the USFS (n = 188) [10], Virginia Department of Game and Inland Fisheries (n = 22), USGS National Water Information System (n = 12), and USGS Leetown Science Center (n = 9). We used a single season of stream and air temperature data because it provided an opportunity for the most spatially extensive analysis of the SAM, but the lack of replication across years added uncertainty to our predictions of future JMMST. Summer stream temperature data collected by these agencies were recorded by digital temperature loggers at ≤ 1-hour intervals at all sites, with the majority of sites recording stream temperatures at 30-minute intervals. After screening stream temperature data for missing records during July, 201 sites (Fig 1) had data suitable for parameterizing models of JMMST.

The JMMST regression models included 32 independent variables representing aspects of climate, hydrogeomorphology, lithology, soil texture, vegetation, and solar radiation hypothesized to explain variation in JMMST (Table 1). These predictors were either obtained directly from available spatial databases or were derived from existing datasets as described in Table 1. Long-term annual and July precipitation and air temperature metrics from the PRISM model were used (PRISM Climate Group, Oregon State University, http://prism.oregonstate.edu, created 10 July 2012; see [27] for methods). Average annual runoff was estimated for each derived watershed from established water balance estimates [28]. Hydrogeomorphic characteristics that were considered in this analysis included watershed area and slope, base flow index, topographic wetness index, and drainage density. Regional geologic maps were classified into five broad classes reflecting acid sensitivity and drainage characteristics, including siliciclastic, argillic, felsic, mafic, and carbonate lithologies [22]. The percentage of each watershed that contained these lithologic types was computed. Soil percent clay was derived using a horizon thickness-weighted average from available soil survey (SSURGO and STATSGO) databases [29].

**Table 1:** Landscape characteristics used for statistical modeling.

Type	Variable ID	Variable Name	Units	Variable Description	Reference
Climate	PPTANN	Average annual precipitation	m	PRISM 30-year normal (1981–2000) average annual precipitation	http://www.prism.oregonstate.edu/normals
	PPTJUL	Average July precipitation	m	PRISM 30-year normal (1981–2000) average July precipitation	http://www.prism.oregonstate.edu/normals
	TANN	Average annual temperature	degree C	PRISM 30-year normal (1981–2000) average annual temperature	http://www.prism.oregonstate.edu/normals
	JMMAT	July 2012 mean daily maximum air temperature	degree C	PRISM mean daily maximum temperature for the month of July, 2012	Christopher Daly, Oregon State University personal communication February 27, 2014
	RUNOFF	Average annual runoff	m	USGS long term (1971–2000) average water balance estimates, determined as precipitation minus evapotranspiration	McCabe and Wolock [28]
Geomorphology	WSAREA	Watershed area	km^2^	Contributing drainage area derived from digital elevation data	Jenson and Domingue [32]
	SLOPE	Watershed slope	degree	Watershed average slope derived from digital elevation data	Burrough and McDonnell [33]
	BFI	Base flow index	Unitless	Ratio of base flow to total flow	Wolock [34]
	TWI	Topographic wetness index	Unitless	The propensity of a soil location to become water saturated according to contributing area (a) and local slope (β): TWI = ln (a/tanβ). Slopes equal to zero were set to 0.001 to avoid division by zero	Beven and Kirkby [35]
	DRAINDENS	Drainage density	m^-1^	Ratio of stream length to watershed area	http://www.horizon-systems.com/NHDPlus/NHDPlusV2_home.php
Lithology	LITHSIL	Siliciclastic lithology	%	Sedimentary rocks primarily composed of silicate minerals (e.g. sandstone; cf., Sullivan et al. [22])	http://mrdata.usgs.gov/geology/state
	LITHARG	Argillic lithology	%	Sedimentary rocks characterized by clay minerals (e.g. shale; cf., Sullivan et al. [22])	http://mrdata.usgs.gov/geology/state/
	LITHFEL	Felsic lithology	%	Igneous rocks rich in feldspar and quartz (e.g. granite; cf., Sullivan et al. [22])	http://mrdata.usgs.gov/geology/state/
	LITHMAF	Mafic lithology	%	Igneous rocks rich in magnesium and iron (e.g. basalt; cf., Sullivan et al. [22])	http://mrdata.usgs.gov/geology/state
	LITHCAR	Carbonate lithology	%	Sedimentary rocks primarily composed of carbonate minerals (e.g. limestone; cf., Sullivan et al. [22])	http://mrdata.usgs.gov/geology/state
Soils	SOILCLAY	Soil percent clay	%	Aspect of soil texture	Soil Survey Staff [36]
Vegetation	FOREST	Forest cover	%	Deciduous, coniferous, or mixed forest type specified in NLCD 2006 (code = 41, 42, or 43)	Fry et al. [30]
	FORESTRIP	Forest cover in riparian zone	%	Deciduous, coniferous, or mixed forest type specified in NLCD 2006 (code = 41, 42, or 43) within area adjacent to streams	Fry et al. [30]
	GRASS	Grassland cover	%	Grassland/herbaceous cover specified in NLCD 2006 (code = 71)	Fry et al. [30]
	GRASSRIP	Grassland cover in riparian zone	%	Grassland/herbaceous cover specified in NLCD 2006 (code = 71) within area adjacent to streams	Fry et al. [30]
	CC	Forest canopy cover	%	Average percent cover of tree canopy in each 30 m grid cell	http://www.landfire.gov/vegetation.php
	CCRIP	Forest canopy cover in riparian zone	%	Average percent cover of tree canopy in each 30 m grid cell within area adjacent to streams	http://www.landfire.gov/vegetation.php
	EVH	Vegetation height	m	Height of the dominant vegetation in a 30 m grid cell	http://www.landfire.gov/vegetation.php
	EVHRIP	Vegetation height in riparian zone	m	Height of the dominant vegetation in a 30 m grid cell within area adjacent to streams	http://www.landfire.gov/vegetation.php
Solar Radiation	SOL57	Solar radiation in watershed	Wh/m^2^	Total incoming solar radiation during the months of June, July and August	Fu and Rich [31]
	SOL57FST	Solar radiation in the watershed divided by forest cover	Wh/m^2^	Calculated as SOL57 divided by FOREST to adjust solar radiation by the extent of forest land cover	Fry et al. [30], Fu and Rich [31]
	SOL57CC	Solar radiation in the watershed divided by canopy cover	Wh/m^2^	Calculated as SOL57 divided by CC to adjust solar radiation by the extent of forest canopy cover	Fu and Rich [31]; http://www.landfire.gov/vegetation.php
	SOL57EVH	Solar radiation in the watershed divided by existing vegetation height	Wh/m^2^	Calculated as SOL57 divided by EVH to adjust solar radiation by the height of existing vegetation	Fu and Rich [31]; http://www.landfire.gov/vegetation.php
	SOL57RIP	Solar radiation in riparian zone	Wh/m^2^	Total incoming solar radiation to area adjacent to streams during the months of June, July and August	Fu and Rich [31]
	SOL57FSTRIP	Solar radiation in riparian zone divided by forest cover in riparian zone	Wh/m^2^	Calculated as SOL57RIP divided by FORESTRIP to adjust riparian solar radiation by the extent of forest land cover in the riparian zone	Fry et al. [30], Fu and Rich [31]
	SOL57CCRIP	Solar radiation in riparian zone divided by canopy cover in riparian zone	Wh/m^2^	Calculated as SOL57RIP divided by CCRIP to adjust riparian solar radiation by the extent of forest canopy cover in the riparian zone	Fu and Rich [31]; http://www.landfire.gov/vegetation.php
	SOL57EVHRIP	Solar radiation in riparian zone divided by existing vegetation height in riparian zone	Wh/m^2^	Calculated as SOL57RIP divided by EVHRIP to adjust riparian solar radiation by the height of existing vegetation in the riparian zone	Fu and Rich [31]; http://www.landfire.gov/vegetation.php

All variables were calculated as watershed average or percentage, with the exception of drainage density and watershed area.

Aspects of vegetation type, cover, and height were derived from the 2006 National Land Cover Dataset (NLCD) and the LANDFIRE database [30]. The amount of incoming solar radiation to the watershed and riparian zone was determined using a simulation model that considered seasonal solar zenith angles and terrain shading [31]. Estimates of gross solar radiative flux were conditioned by the percent of forest cover, average canopy cover, and vegetation height to represent the extent to which vegetation may reduce total solar radiation (Table 1).

We evaluated all possible combinations of independent variables in models of JMMST. The model with the lowest Akaike Information Criterion (AIC) value was selected as the most plausible model, given the available data, and all independent variables were required for inclusion to be significant at p < 0.05. This model was then used to predict contemporary JMMST at each reach across the SAM stream network.

#### Future stream temperature

An assessment of potential increases in stream temperature associated with atmospheric warming should consider spatial variation in air and stream temperature relationships. Some stream reaches are strongly buffered from the physical processes that influence air temperature, whereas others are highly sensitive to those processes. Site-specific correlations between air and stream temperatures are therefore useful for understanding spatial variation in stream temperature responses to climate change. Consequently, we first characterized air and stream temperature correlations for the summer period (June 1 to August 31) for stream temperature measurement sites with available data (n = 191). We used air temperatures derived from 800-m resolution PRISM data to calculate watershed averages of maximum daily air temperature (MDAT) for each day between June 1 and August 31, 2012. We validated PRISM-derived estimates of MDAT against MDAT calculated from continuous (≤ 1 hr intervals) air temperature measurements made at 180 of the USFS stream temperature monitoring sites [10]. PRISM data were in close agreement with field measurements of MDAT (linear regression; mean r^2^ = 0.90).

We performed linear regressions of MDAT on maximum daily stream water temperature (MDST) data for June 1 to August 31, 2012 for each stream temperature measurement site. The resulting regression slope coefficients (*β*), which represent the unit change in MDST associated with a 1°C change in MDAT, were highly variable across sites, ranging from 0.02 to 0.96°C, with a median of 0.43°C. Variation in *β* among sites reflects spatial differences in the strength of coupling between processes controlling air and stream temperatures. Understanding spatial variation in *β* provides a means for predicting JMMST responses associated with future increases in air temperature.

To identify landscape characteristics associated with variation in *β*, we developed multiple linear regression models to predict *β* from the suite of hydrogeomorphology, lithology, soil texture, vegetation, and solar radiation variables listed in Table 1. Initial linear models consistently overestimated *β* for sites where observed *β* < 0.275, but provided reasonable predictions where observed *β* ≥ 0.275. The poor performance of linear models for sites with low *β* most likely reflects a limitation of watershed-scale variables to detect site-level processes controlling stream temperature. For example, localized hyporheic exchange and groundwater inputs can buffer stream temperature from the influence of processes that control air temperature, resulting in low values for *β*. Consequently, we used only sites with observed *β* ≥ 0.275 (hereafter “high-sensitivity sites”; n = 160) to parameterize subsequent linear regression models. For sites where observed *β* < 0.275 (hereafter “low-sensitivity sites”), we assumed that responses of stream temperature to increased air temperature would be negligible.

In order to develop a predictive model for identifying high-sensitivity and low-sensitivity stream reaches, we performed multiple logistic regression (Fig 2b). Low-sensitivity sites (observed *β* < 0.275) were given a value of 0 and high-sensitivity sites (observed *β* ≥ 0.275) were given a value of 1. Independent variables in logistic regression models included hydrogeomorphology, lithology, soil texture, vegetation, and solar radiation variables listed in Table 1. We used AIC to evaluate competing models, and all independent variables were required to be significant at p<0.05. We used a probability cutoff of 0.50, estimated from the final logistic regression model, to categorize sites as having either high sensitivity or low sensitivity.

The parameterized logistic regression and multiple linear regression models were used to estimate potential stream temperature responses correlated with increased air temperature for all stream reaches in the SAM study area according to the following steps. First, we used the logistic regression model to categorize stream reaches as having either high or low sensitivity to air temperature (Fig 2b). For high-sensitivity reaches, we then used the multiple linear regression model to predict the slope coefficient *β* (Fig 2c), which represents the unit change in MDST associated with a 1°C change in MDAT. We then predicted changes in contemporary JMMST according to MDAT increases of 2 and 4°C by multiplying these air temperature increases by the slope coefficient *β*, and adding their product to the contemporary JMMST estimate (Fig 2d).

We analyzed the potential effects of air temperature increases of 2 and 4°C, based on downscaled regional climate projections from 16 general circulation models (GCMs) assuming the A2 emissions scenario [37, 38]. These models estimate changes in average annual air temperature of +2.1°C to +5.3°C (median 4.1), by the mid-2080s. We chose increases in July mean MDAT of 2 and 4°C above contemporary values as reasonable approximations of increases to mid- and end-of-century MDAT, respectively.

### Acid neutralizing capacity (ANC) estimates

We modified a previously published model of stream water ANC (cf., [23]) to identify locations in the SAM where ANC may cause biological impairment. This modeling process involved two steps. The first step used a binomial model to categorize stream reaches as having either high ANC (> 300 μeq/L) or low ANC (< 300 μeq/L). In the second step, low-ANC reaches were entered into linear models to estimate reach-specific ANC values; high-ANC reaches are well above ANC levels considered potentially harmful for aquatic biota and were not considered further. We modified the original linear ANC model for low-ANC reaches [23] to correct for an apparent bias in which the model under-predicted ANC values above 75 μeq/L and over-predicted those below 75 μeq/L. These results were adjusted for this apparent bias as described in the Supporting Information (S1 Fig).

### Delineating suitable habitat for acid-sensitive coldwater species

The JMMST threshold was selected to represent upper limits to the preferred temperature range for the coldwater species guild [39–41], including salmonid (e.g., brook trout) and cottid (e.g., mottled sculpin [*Cottus bairdi*] and slimy sculpin [*C*. *cognatus*]) fishes. The ANC threshold was selected based on evidence of substantial negative biological effects on stream macroinvertebrate and fish species at ANC < 50 μeq/L [42]. We estimated the extent of potential habitat loss by comparing the stream length of suitable habitat for acid-sensitive coldwater aquatic species under contemporary conditions with that associated with air temperature increases of 2 and 4°C. We summarized changes in the length of suitable stream habitat within each ranger district and NF.

## Results

### Contemporary Stream Temperature Prediction

#### July Mean Daily Maximum Stream Water Temperature

**Fig 3:**
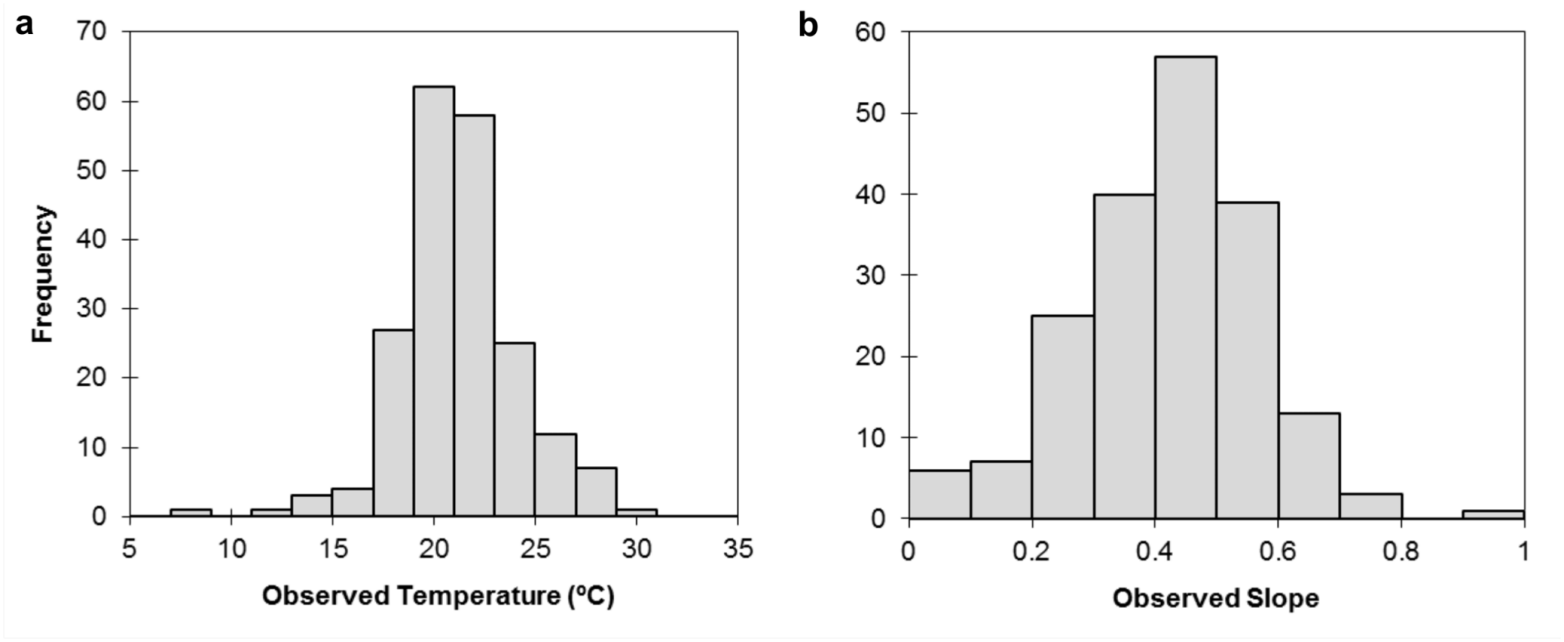
Distribution of observed a) July mean daily maximum stream water temperature (JMMST; n = 201) and b) slope of the relationship between daily maximum air and water temperature from June 1, 2012 to August 31, 2012 (n = 191).

**Fig 4:**
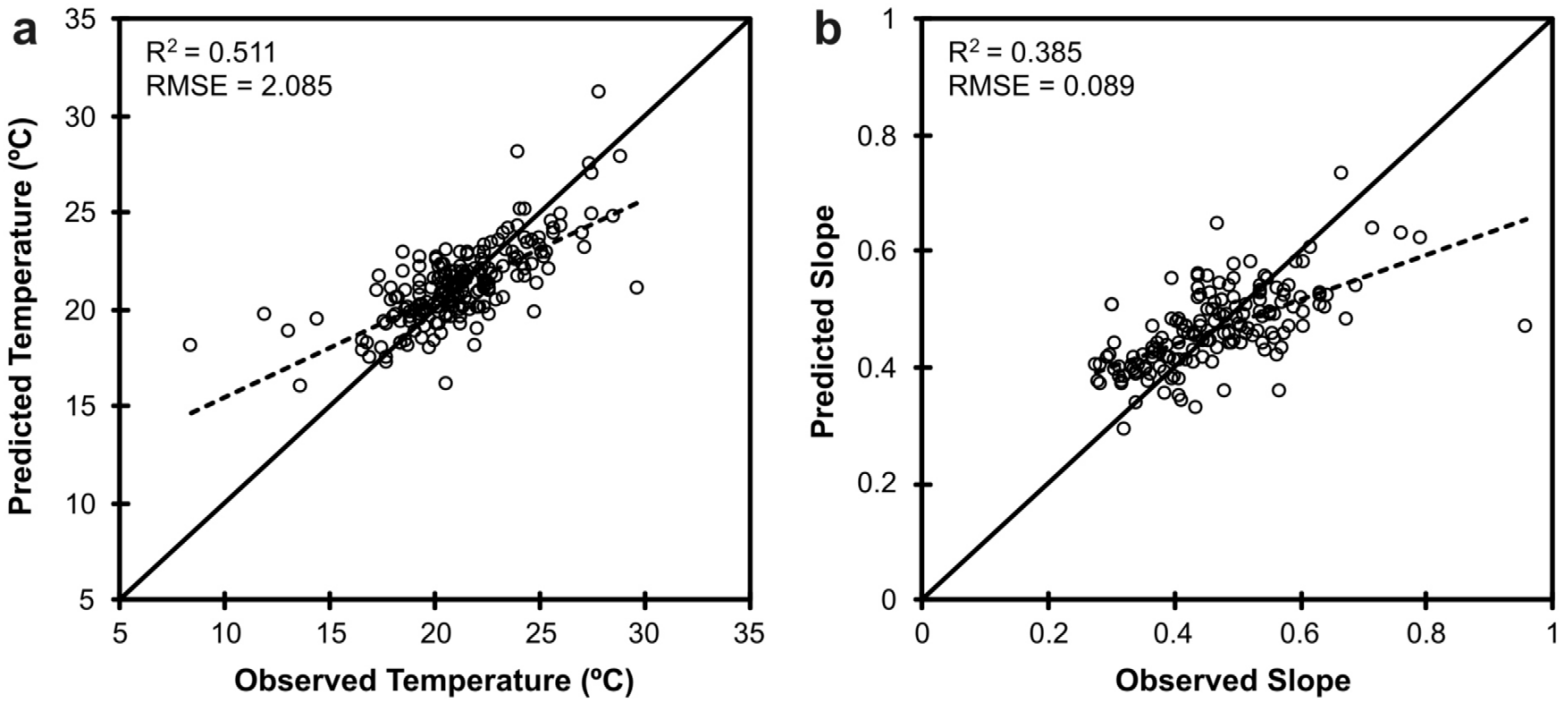
Scatterplots of predicted vs. observed a) July mean daily maximum stream water temperature (JMMST; n = 201) and b) slope of the air-water relation based on logistic and multiple linear regression models (n = 191). The solid black line indicates the 1:1 relationship between predicted and observed values. The dashed line indicates the best fit regression of predicted and observed values.

The distribution of observed JMMST at measurement sites (n = 201) was approximately normal with a mean of 21.2°C (SD = 2.9°C; Fig 3a). The best predictive model of JMMST included, in order of decreasing influence, July mean MDAT, watershed area, percent carbonate lithology, base flow index, percent riparian canopy cover, average July precipitation, and siliciclastic lithology as explanatory variables. The JMMST model had r^2^ = 0.51 and root mean-squared error (RMSE) = 2.09 (Fig 4a). Sites having higher July mean MDAT, watershed area, and July precipitation tended to have higher JMMST. Coefficient estimates for each variable included in the final JMMST model are provided in Table 2.

**Table 2:** Coefficients and descriptive statistics associated with the multiple linear regression model for predicting July mean daily maximum stream water temperature (JMMST).

Variable Name	Variable ID	Coefficient	Standardized Coefficient	p-value	Variance Inflation Factor (VIF)
July 2012 mean daily maximum air temperature	JMMAT	0.8714	0.4977	< 0.0001	1.28
Watershed area	WSAREA	0.0225	0.3845	< 0.0001	1.07
Carbonate lithology	LITHCAR	-0.0727	-0.3519	< 0.0001	1.25
Base flow index	BFI	-0.0934	-0.3120	< 0.0001	1.37
Forest canopy cover in riparian zone	CCRIP	-0.0508	-0.2621	< 0.0001	1.31
Average July precipitation	PPTJUL	0.0254	0.1697	0.005	1.41
Siliciclastic lithology	LITHSIL	-0.0153	-0.1557	0.008	1.34

### Future stream temperatures

#### Air and stream temperature relationships

The distribution of slope coefficients (*β*) from regressions of observed MDST on MDATs for the June 1 through August 30 summer period was approximately normal with a mean of 0.42 (SD = 0.15; Fig 3b). Of the 191 stream temperature measurement sites, 31 had slope coefficients < 0.275 and were considered to be sites with low sensitivity to air temperature change. The remaining 160 sites had slope coefficients ≥ 0.275 and were considered to be sites having high sensitivity to air temperature change.

The best logistic regression model with which to predict stream temperature sensitivity (i.e., high vs. low) included watershed area, percent carbonate lithology, and riparian vegetation height as significant predictor variables. Sites with taller riparian vegetation, a greater percentage of carbonate lithology, and smaller watershed area were more likely to have low sensitivity to changes in air temperature. This model correctly classified 98.1% (157/160) of the high-sensitivity sites and 32.3% (10/31) of the low-sensitivity sites, for an overall correct classification rate of 87.4%. Coefficient estimates for each variable included in the final logistic regression model are provided in Table 3.

**Table 3:** Coefficients and descriptive statistics associated with the logistic regression model for predicting maximum daily stream water temperature (MDST) sensitivity (high/low) to changes in maximum daily air temperature (MDAT).

Variable Name	Variable ID	Coefficient	Standardized Coefficient	p-value
Watershed area	WSAREA	-0.053	-1.567	0.017
Carbonate lithology	LITHCAR	0.074	0.601	< 0.001
Vegetation height in riparian zone	EVHRIP	0.145	0.505	0.000

For stream temperature measurement sites with observed high sensitivity to air temperature (i.e., slope coefficients ≥ 0.275), a multiple linear regression model that included base flow index, watershed area, percent siliciclastic lithology, percent carbonate lithology, and topographic wetness provided the best predictions of site-specific slope coefficient values (r^2^ = 0.39, RMSE = 0.09; Fig 4b). Sites having lower values for base flow index, percent siliciclastic lithology, and percent carbonate lithology, and higher values for watershed area and topographic wetness tended to have larger slope coefficients. Coefficient estimates for each variable included in the final regression model are provided in Table 4.

**Table 4:** Coefficients and descriptive statistics associated with the multiple linear regression model^1^ for continuous estimates of the strength of maximum daily stream temperature (MDST) and maximum daily air temperature (MDAT) correlations.

Variable Name	Variable ID	Value	Standardized Value	p-value	Variance Inflation Factor (VIF)
Base flow index	BFI	-0.0051	-0.4546	< 0.0001	1.40
Watershed area	WSAREA	0.0006	0.3279	< 0.0001	1.06
Siliciclastic lithology	LITHSIL	-0.0011	-0.2905	< 0.0001	1.25
Topographic wetness index	TWI	0.0721	0.2514	< 0.0001	1.18
Carbonate Lithology	LITHCAR	-0.0024	-0.2150	0.0020	1.18

^1^ This model was only applied to reaches for which MDST was considered to have high sensitivity to increases in MDAT based on the logistic regression model (Table 3).

### Regional habitat suitability

#### Contemporary stream temperature and ANC

Our JMMST model predictions suggested that the amount of stream habitat suitable for coldwater species (i.e., JMMST < 20°C) under contemporary conditions is moderate to low as a percentage of total stream length contained in each of the SAM NFs (mean = 26%, range = 3–49%; Table 5). The proportion of stream length suitable for coldwater species varied according to JMMST thresholds of 18°C (mean = 6%, range = 0–15%; S1 Table) and 22°C (mean = 59%, range = 33–83%; S2 Table). Pisgah and Nantahala NFs contained the most coldwater habitat both in terms of absolute stream length having JMMST < 20°C (approximately 4000 km each) and as a percentage of total stream length within each NF (nearly 50%).

**Table 5:** Length of stream and percentage of total stream length that was predicted to be: too warm (> 20°C) during July, too acidic (ANC < 50 μeq/L), or suitable (ANC > 50 μeq/L and T < 20°C) for sensitive species.

National Forest/ Ranger District	Total Stream Length	Temp > 20°C		ANC < 50 μeq/L		Temp > 20°C and ANC < 50 μeq/L		Suitable Habitat	
	km	km	%	km	%	km	%	km	%
**George Washington**									
James River	2,505	2,395	95.6	59	2.4	17	0.7	68	2.7
Lee	1,904	1,725	90.6	114	6.0	88	4.6	153	8.0
North River	3,857	3,607	93.5	475	12.3	315	8.2	90	2.3
Pedlar	1,678	1,408	83.9	174	10.4	92	5.5	187	11.2
Warm Springs	2,147	1,875	87.3	118	5.5	35	1.6	189	8.8
*TOTAL*	*12*,*090*	*11*,*009*	*91*.*1*	*939*	*7*.*8*	*546*	*4*.*5*	*687*	*5*.*7*
**Jefferson**									
Clinch	1,851	1,782	96.3	219	11.8	209	11.3	59	3.2
Eastern Divide	5,236	3,654	69.8	559	10.7	72	1.4	1,095	20.9
Glenwood	1,139	1,014	89.1	97	8.5	94	8.2	122	10.7
Mt. Rogers	2,630	913	34.7	293	11.1	14	0.5	1,438	54.7
*TOTAL*	*10*,*856*	*7*,*363*	*67*.*8*	*1*,*168*	*10*.*8*	*389*	*3*.*6*	*2*,*714*	*25*.*0*
**Cherokee**									
Nolichucky	3,927	2,878	73.3	222	5.6	87	2.2	914	23.3
Ocoee	1,696	1,618	95.4	76	4.5	41	2.4	44	2.6
Tellico	1,507	1,177	78.1	217	14.4	32	2.1	145	9.6
Watauga	4,053	2,208	54.5	284	7.0	31	0.8	1,593	39.3
*TOTAL*	*11*,*183*	*7*,*881*	*70*.*5*	*798*	*7*.*1*	*191*	*1*.*7*	*2*,*695*	*24*.*1*
**Pisgah**									
Appalachian	3,968	2,302	58.0	315	7.9	70	1.8	1,421	35.8
Grandfather	2,330	1,227	52.7	138	5.9	9	0.4	974	41.8
Pisgah	1,706	571	33.5	115	6.7	0	0.0	1,020	59.8
*TOTAL*	*8*,*004*	*4*,*100*	*51*.*2*	*568*	*7*.*1*	*79*	*1*.*0*	*3*,*415*	*42*.*7*
**Nantahala**									
Cheoah	1,768	957	54.1	355	20.1	15	0.8	471	26.6
Nantahala-Highlands	1,638	406	24.8	111	6.8	0	0.0	1,121	68.4
Nantahala-Wayah	2,573	1,265	49.2	156	6.1	0	0.0	1,152	44.8
Tusquitee	2,999	2,278	76.0	105	3.5	19	0.6	634	21.1
*TOTAL*	*8*,*978*	*4*,*906*	*54*.*6*	*728*	*8*.*1*	*34*	*0*.*4*	*3*,*378*	*37*.*6*
**Chattahoochee**									
Blue Ridge	3,285	2,898	88.2	1	0.0	0	0.0	386	11.8
Chattooga River	2,371	1,888	79.6	42	1.8	2	0.1	444	18.7
Conasauga	3,362	3,091	91.9	0	0.0	0	0.0	271	8.1
*TOTAL*	*9*,*017*	*7*,*877*	*87*.*4*	*42*	*0*.*5*	*2*	*0*.*0*	*1*,*101*	*12*.*2*
**Sumter**									
Andrew Pickens	906	881	97.2	0	0.0	0	0.0	25	2.8
*TOTAL*	*906*	*881*	*97*.*2*	*0*	*0*.*0*	*0*	*0*.*0*	*25*	*2*.*8*
***Total Stream Length***	***61*,*035***	***44*,*017***		***4*,*243***		***1*,*241***		***14*,*015***	
***Average %***			***72*.*8***		***7*.*0***		***2*.*2***		***22*.*4***

ANC models predicted that biological impairment for acid-sensitive species occurs in all NFs included in this study, with the exception of Sumter NF where no stream reaches were predicted to have ANC < 50 μeq/L (Table 5). The percentage of total stream length having ANC < 50 μeq/L within each NF (mean = 6%, range = 0–11%) was much less than that predicted to exceed the temperature threshold for coldwater species (mean = 74%, range = 51–97%). Stream reaches that both exceeded the temperature threshold of 20°C and fell below the ANC threshold of 50 μeq/L were rare, accounting for only 2% (range = 0–5%) of the total stream length contained in each NF. Consequently, most stream reaches predicted to have ANC < 50 μeq/L occurred in locations with otherwise suitable thermal habitat for coldwater species under contemporary conditions. Assuming that ANC < 50 μeq/L renders this habitat inhospitable for acid-sensitive aquatic species, low ANC precluded use of approximately 16% (range = 0–37%) of the length of suitable thermal habitat for coldwater species within each NF. Model projections for stream temperature and ANC thresholds for all NFs within the SAM are provided in S2 Fig. There is no clear spatial pattern of habitat suitability with respect to stream temperature and ANC conditions throughout the study area. July mean daily maximum air temperature (JMMAT), which is the strongest predictor of JMMST, are not well correlated with latitude (r^2^ = 0.009). However, JMMAT is strongly correlated with elevation (r^2^ = 0.917). Elevation is heterogeneous in this mountainous region (Table 6, Fig 1), and overrides the influence of lower latitude and associated increased solar radiation inputs to provide a greater proportion of suitable coldwater habitat in the south as compared with more northern stream networks.

**Table 6:** Range and average elevation (m) of all watersheds contained within each national forest comprising the study region.

National Forest	Minimum Elevation	Average Elevation	Maximum Elevation
George Washington	220	705	1200
Jefferson	249	798	1554
Cherokee	250	751	1497
Pisgah	380	1010	1775
Nantahala	431	943	1689
Chattahoochee	193	642	1267
Sumter	276	554	1030

#### Habitat loss from stream warming

The amount of suitable stream habitat for acid-sensitive coldwater species is predicted to decrease in all SAM NFs with future increases in air temperature (Table 7). With a 2°C increase in July mean MDAT, the predicted mean stream temperature response was an increase of 0.76°C above contemporary JMMST. This increase resulted in an approximately 6% reduction (range = 2–12%) of the total stream length within each NF with predicted JMMST < 20°C (Table 7). A 4°C increase in MDAT produced a mean stream temperature increase of 1.52°C above contemporary JMMST, and an approximately 10% reduction in total stream length within each NF with predicted JMMST < 20°C. These changes in stream temperature and thermal habitat loss incorporate the predictions of the logistic regression model that we used to classify stream reaches as having either low or high sensitivity to air temperature. We estimated that approximately 27% of the SAM stream network has low sensitivity to air temperature, and we therefore assumed no stream temperature warming in those reaches in response to increases in MDATs.

**Table 7:** Length and percentage of suitable stream habitat for acid- and thermally sensitive species in each national forest and ranger district within the study region.

National Forest / Ranger District		Suitable Habitat							
	Total Stream Length	Contemporary Air Temp		+2°C Δ Air Temp			+4°C Δ Air Temp		
	km	km	%	km	%	Δ from Ambient (%)	km	%	Δ from Ambient (%)
**George Washington**									
James River	2,505	68	2.7	35	1.4	-1.3	32	1.3	-1.4
Lee	1,904	153	8.0	137	7.2	-0.9	137	7.2	-0.9
North River	3,857	90	2.3	13	0.3	-2.0	8	0.2	-2.1
Pedlar	1,678	187	11.2	168	10.0	-1.2	166	9.9	-1.3
Warm Springs	2,147	189	8.8	65	3.1	-5.7	36	1.7	-7.1
*TOTAL*	*12*,*090*	*687*	*5*.*7*	*418*	*3*.*5*	*-2*.*2*	*379*	*3*.*1*	*-2*.*6*
**Jefferson**									
Clinch	1,851	59	3.2	59	3.2	0.0	59	3.2	0.0
Eastern Divide	5,236	1,095	20.9	703	13.4	-7.5	551	10.5	-10.4
Glenwood	1,139	122	10.7	115	10.1	-0.6	115	10.1	-0.6
Mt. Rogers	2,630	1,438	54.7	1,146	43.6	-11.1	923	35.1	-19.6
*TOTAL*	*10*,*856*	*2*,*714*	*25*.*0*	*2*,*023*	*18*.*6*	*-6*.*4*	*1*,*649*	*15*.*2*	*-9*.*8*
**Cherokee**									
Nolichucky	3,927	914	23.3	735	18.7	-4.6	672	17.1	-6.2
Ocoee	1,696	44	2.6	31	1.8	-0.7	30	1.8	-0.8
Tellico	1,507	145	9.6	111	7.4	-2.2	100	6.6	-3.0
Watauga	4,053	1,593	39.3	1,331	32.8	-6.5	1,231	30.4	-8.9
*TOTAL*	*11*,*183*	*2*,*695*	*24*.*1*	*2*,*208*	*19*.*7*	*-4*.*4*	*2*,*033*	*18*.*2*	*-5*.*9*
**Pisgah**									
Appalachian	3,968	1,421	35.8	975	24.6	-11.2	688	17.3	-18.5
Grandfather	2,330	974	41.8	668	28.7	-13.1	486	20.8	-21.0
Pisgah	1,706	1,020	59.8	808	47.3	-12.4	575	33.7	-26.1
*TOTAL*	*8*,*004*	*3*,*415*	*42*.*7*	*2*,*451*	*30*.*6*	*-12*.*0*	*1*,*748*	*21*.*8*	*-20*.*8*
**Nantahala**									
Cheoah	1,768	471	26.6	254	14.3	-12.3	142	8.0	-18.6
Nantahala-Highlands	1,638	1,121	68.4	881	53.8	-14.6	637	38.9	-29.5
Nantahala-Wayah	2,573	1,152	44.8	843	32.8	-12.0	596	23.2	-21.6
Tusquitee	2,999	634	21.1	487	16.2	-4.9	398	13.3	-7.9
*TOTAL*	*8*,*978*	*3*,*378*	*37*.*6*	*2*,*465*	*27*.*5*	*-10*.*2*	*1*,*772*	*19*.*7*	*-17*.*9*
**Chattahoochee**									
Blue Ridge	3,285	386	11.8	194	5.9	-5.8	134	4.1	-7.7
Chattooga River	2,371	444	18.7	276	11.6	-7.1	190	8.0	-10.7
Conasauga	3,362	271	8.1	259	7.7	-0.3	259	7.7	-0.3
*TOTAL*	*9*,*017*	*1*,*101*	*12*.*2*	*730*	*8*.*1*	*-4*.*1*	*583*	*6*.*5*	*-5*.*7*
**Sumter**									
Andrew Pickens	906	25	2.8	4	0.5	-2.3	4	0.5	-2.3
*TOTAL*	*906*	*25*	*2*.*8*	*4*	*0*.*5*	*-2*.*3*	*4*	*0*.*5*	*-2*.*3*

Predicted results are shown for contemporary July mean daily maximum air temperature (MDAT) conditions along with potential future increases in ambient July MDAT of 2°C and 4°C.

**Fig 5:**
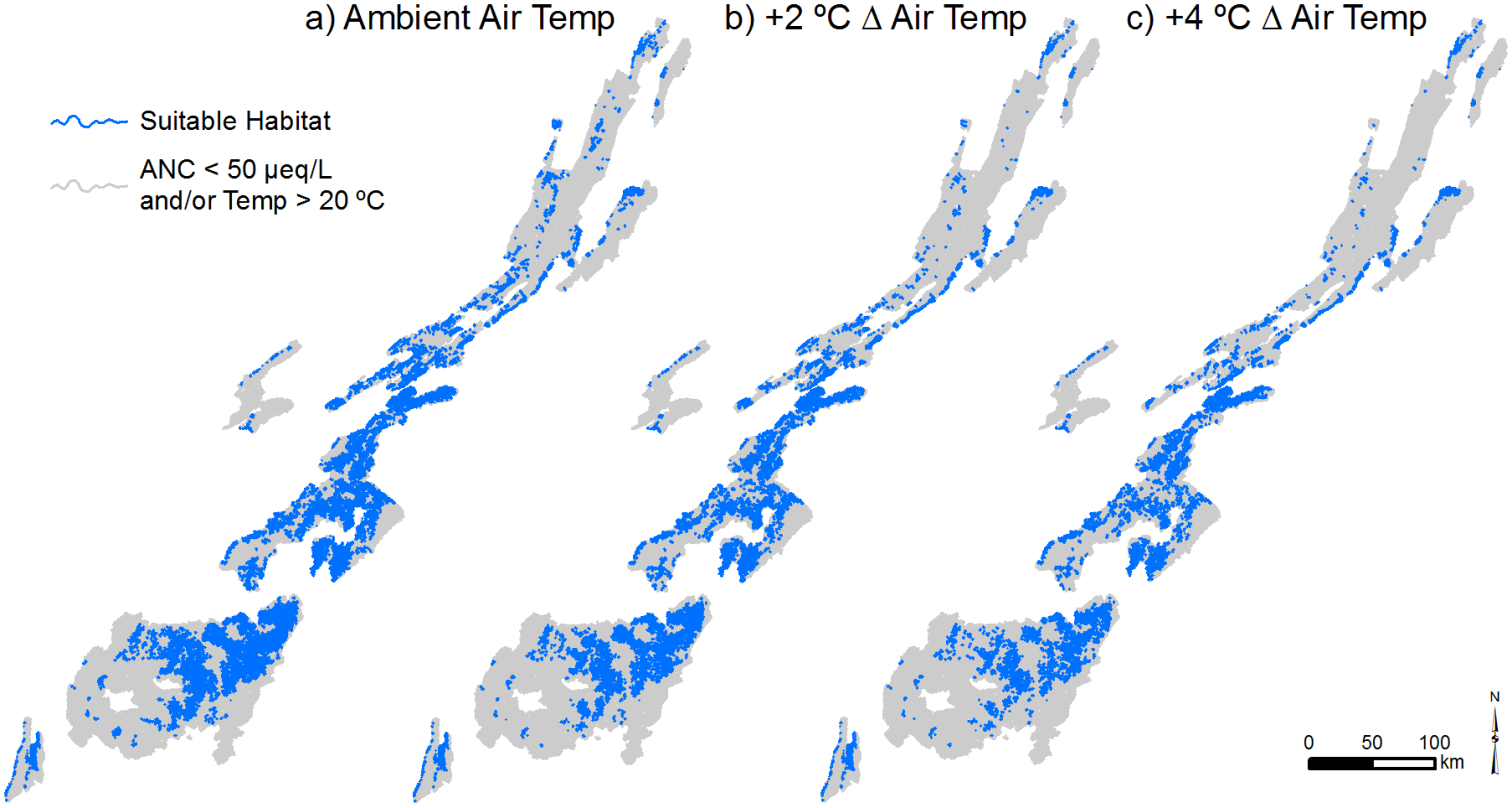
Locations of suitable and unsuitable stream habitat throughout the study region, based on broadly applicable thresholds for acid- and thermally sensitive species. Suitable stream habitat is shown in blue. Streams with ANC < 50 μeq/L and/or temperature > 20°C are considered unsuitable and are shown in gray. Modeled habitat suitability results are shown for a) current July mean daily maximum air temperature (MDAT), and future increases of b) 2°C and c) 4°C.

**Fig 6:**
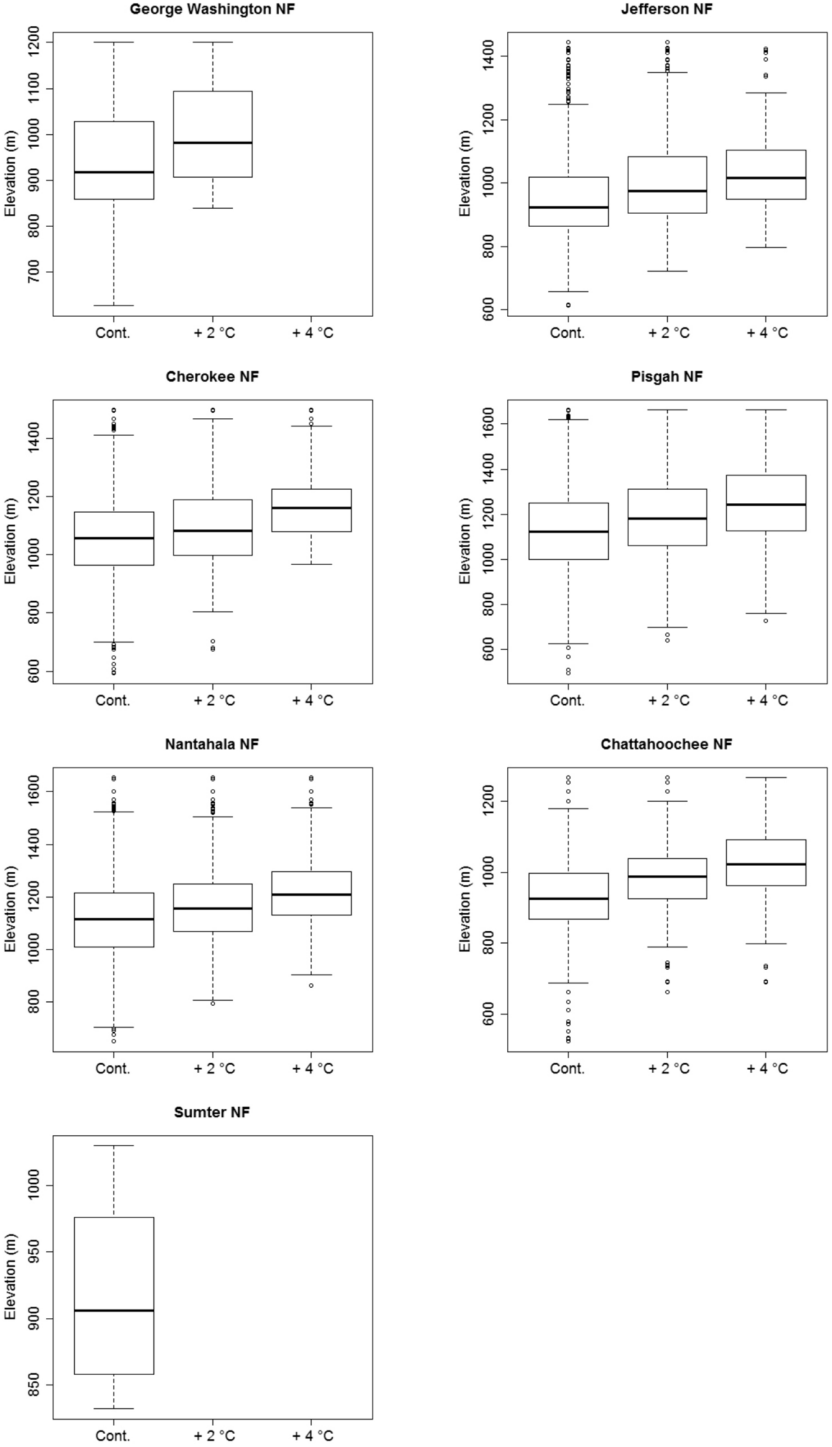
Distribution of average watershed elevation associated with individual stream segments considered to be suitable habitat for coldwater acid-sensitive species according to stream water temperature and ANC thresholds of 20°C and 50 μeq/L, respectively. Results are shown for the three scenarios of July mean maximum daily air temperature (MDAT) considered in this study, including contemporary (Cont.) and July mean MDAT change of + 2°C and + 4°C. Only stream segments determined to have “high” sensitivity to summer period (June 1 –August 31) MDAT increases according to logistic regression results (Fig 2b) are included. Only scenarios for which suitable habitat existed are shown.

For the majority of NFs, the largest reductions in stream length having suitable thermal habitat are predicted to occur in response to a 2°C increase in MDAT (Table 7). Losses of suitable thermal habitat corresponding with air temperature increases between 2 and 4°C above contemporary conditions are approximately half of those predicted for air temperature increases of 2°C above contemporary conditions (Table 7). Pisgah and Nantahala NFs are exceptions to this pattern, however, and losses of thermal habitat in these forests are not greatly diminished above a 2°C increase in MDAT (Table 7). These NFs also are expected to experience the greatest overall reductions in suitable thermal habitat. Thermal habitat losses associated with a 2°C increase in MDAT are predicted to be close to 900 km, or approximately 10% of total stream length within each of these NFs. The amount of thermal habitat loss in these NFs is predicted to increase to approximately 1600 km each with a 4°C increase in MDAT. This represents 21 and 18% of the total stream length in Pisgah and Nantahala NFs, respectively (Table 7). In general, the effect of incremental stream warming is a predicted contraction of suitable thermal habitat towards low-order headwater locations (Fig 5). The distribution of suitable habitat for acid-sensitive coldwater species was projected to shift from lower to higher elevations associated with increased July mean MDAT across all NFs (Fig 6).

**Fig 7:**
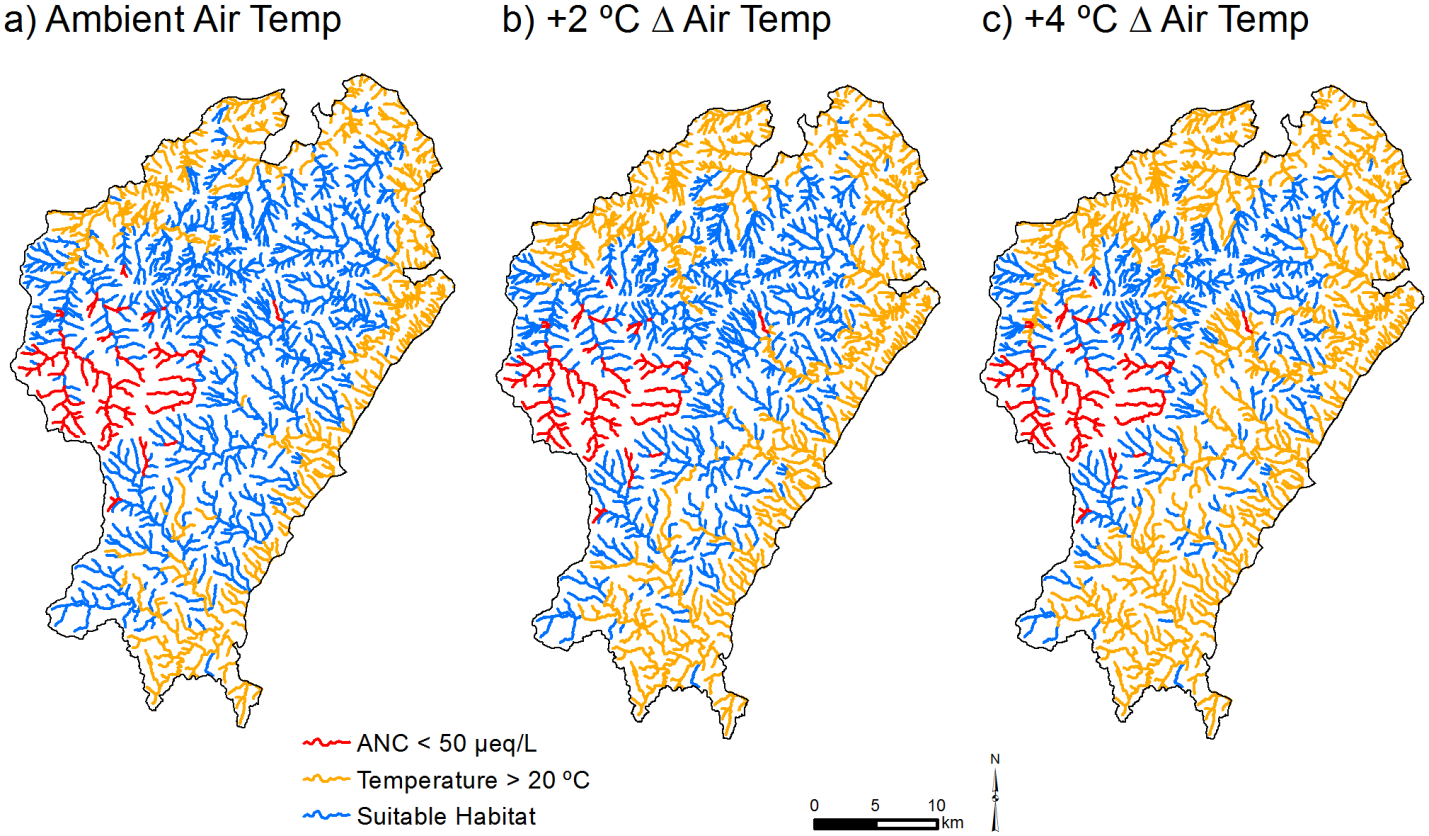
Streams located in the Pisgah Ranger District of the Pisgah National Forest, which comprises 2.8% of the stream length in the study region, having predicted ANC < 50 μeq/L (red), temperature > 20°C (orange), or suitable habitat with respect to both ANC and temperature (blue). Modeled habitat suitability results are shown for a) ambient July mean maximum daily air temperature (MDAT), and future increases in July mean MDAT of b) 2°C, and c) 4°C. The suitable stream habitat under contemporary July MDAT that is located in the west-central portion of the ranger district is predominantly located in the Shining Rock Wilderness.

Fig 7 illustrates detailed model projections for stream temperature and ANC above and below thresholds in portions of Pisgah NF under contemporary and future climate scenarios. In portions of the stream network where ANC is not a concern, stream warming shifts suitable thermal habitat towards higher elevations. In many areas, the shift in suitable thermal habitat advances towards stream reaches that fall below the ANC threshold of 50 μeq/L. The combined effect of low ANC and stream warming is to restrict suitable habitat for acid-sensitive coldwater species to a narrowing band of mid-elevation stream reaches in some portions of the stream network (Fig 7). This band of suitable habitat is virtually eliminated in some areas due to a convergence of stream reaches that have either JMMST > 20°C or ANC < 50 μeq/L (Fig 7).

## Discussion

### Habitat suitability for acid-sensitive coldwater species

Headwater streams typically provide the coldest available habitat within stream networks and are often perceived as potential climate refugia for coldwater species. The relative mobility of many aquatic species may enable populations to track changes in thermal habitat, provided that constraints associated with stream size, steepness, or other barriers do not limit upstream movement [3]. Climate-induced shifts towards headwater streams have been observed in some fish populations in response to contemporary warming [2, 6]. However, our analysis of spatial patterns of stream temperature and acidification in the SAM suggests that species’ distributional shifts to colder, higher elevation habitats can be constrained by acidification of headwater streams. Headwater acidity is expected to persist in some watersheds for decades, even with substantial reductions in atmospheric deposition of S and N [13, 17, 19]. Low rates of mineral base cation weathering combined with release from the soil of previously adsorbed SO_4_^2-^ are the primary causes of this expected delayed recovery of stream ANC conditions in the SAM region [19]. Consequently, managers will need to continue consideration of stream ANC as a potentially important limiting factor for aquatic species in addition to the expected impacts of increasing MDAT on JMMST described here.

A key finding of our analysis is that there is little spatial overlap in streams that are either too warm or too acidic for sensitive aquatic species. Thus, over much of the SAM region, habitat loss from stream warming and acidification will be additive rather than compensatory. In many areas of the SAM, suitable habitat for acid- and thermally sensitive species will shift to the middle portion of existing coldwater reaches that occur below headwaters that have low ANC, and above low elevation reaches where JMMST will likely exceed 20°C. With increases in future air temperature, stream warming is predicted to progress upstream (Fig 7), causing the elimination of coldwater habitat in some branches of the stream network, or encroaching on reaches that are suitably cold but that have low ANC. Thus, with stream warming, we predict an incremental contraction in the extent of mid-elevation stream reaches having both adequate ANC and suitable stream temperature for coldwater species. Suitable stream habitat under ambient July mean MDAT was estimated to be 23% (14,015 km) of total stream length (61,034 km), with a range of 2–68% among ranger districts. Reductions in suitable habitat with a future increase in July mean MDAT of 2°C ranged from almost zero to nearly 15% among ranger districts (6% of total stream length; 2,316 km), with five ranger districts losing more than 800 km of thermally suitable stream length. Losses associated with a 4°C increase in July mean MDAT ranged from almost zero to nearly 30% of stream length among ranger districts, which translates to nearly a 10% reduction (5,850 km) across all stream length.

One of the challenges facing watershed managers is understanding spatial variability in the response of local stream temperature to regional climate projections. Air temperature-elevation relationships are common surrogates for stream temperature when projecting the potential effects of climate warming on stream ecosystems. Nevertheless, a variety of local controls are known to alter relationships between air and stream temperature (e.g., see [10, 43, 44]). Stream temperature forecasts that assume direct correspondence with air temperature tend to overestimate the extent of thermal habitat loss [10]. This occurs mostly because of variability in riparian shading and groundwater contributions among stream watersheds. Consequently, we attempted to account for spatial variation in stream temperature sensitivity by directly modeling the sensitivity of JMMST to changes in MDAT. We observed considerable spatial variation in the sensitivity of stream temperature to increases in air temperature. The mean predicted July maximum stream temperature increase associated with the 4°C increase in July mean MDAT was approximately 1.7°C (i.e., mean stream temperature increase per unit increase in air temperature = 0.42°C). However, paired air and stream temperature records for the study area indicated statistical independence between air and stream temperature at some sites, and a nearly one-to-one relationship at others.

Stream sites having low sensitivity to increased air temperature (air-stream temperature slope coefficient < 0.275) were associated with small watershed area, high percentage area in carbonate lithologies, and tall riparian vegetation. This suggests that small, well-shaded streams with an underlying geology that supports cold groundwater contribution to base flow display the lowest sensitivity to atmospheric warming. At locations where our model misidentified low-sensitivity sites, we likely overestimated the stream temperature response to atmospheric warming. Efforts to improve understanding of geologic and geomorphic factors directly associated with groundwater contributions to base flow will improve climate change assessments [10]. Nevertheless, our approach was useful for identifying many portions of the SAM where stream temperature is not likely to be sensitive to atmospheric warming.

The majority of high-sensitivity stream temperature sites where maximum stream temperatures are likely to increase with atmospheric warming were correctly classified based on logistic regression. Among these sites, stream temperature sensitivity to change in air temperature increased with increasing watershed area and topographic wetness, and decreased with increases in a variety of measures of groundwater contribution to surface flow (i.e., baseflow index, siliciclastic and carbonate lithologies). Longer surface water transport time within large watersheds generally provides more opportunity for water temperature to equilibrate with air temperature. The sign of each predictor was consistent with the hypothesized direction of influence on water temperature and relations between air and water temperature.

Overall, predicted stream temperatures associated with a July mean MDAT increase of 4°C would reduce coldwater habitat throughout the SAM by ~10%, with some NFs experiencing up to 20% reduction. Model results suggested that streams on the Pisgah and Nantahala NFs will be most impacted among SAM forests. These NFs had relatively high proportions of coldwater stream habitat under contemporary conditions, but a high percentage of this coldwater habitat had predicted JMMSTs near the 20°C threshold. Consequently, even small increases in predicted JMMST resulted in relatively large amounts of habitat loss in these NFs as a proportion of total stream length.

### Uncertainty

Regional ANC estimates are more accurate for streams with ANC in the range of biological sensitivity than those that have higher ANC ([23]; S1 Fig). The RMSE for streams with ANC predictions < 150 μeq/L was 36.6 μeq/L, whereas the RMSE was 107.5 μeq/L for the full ANC range [23]. Spatial variation in uncertainty of regional ANC predictions was demonstrated by Povak et al. [23] and was generally lower for streams located on the national forests of interest in our study relative to other portions of the SAM region.

Regional results of JMMST presented here are first approximations generated from data available at the time of this study and are subject to uncertainty derived from multiple sources, including accuracy of predictor variable datasets, use of a single year of data to represent contemporary stream temperature conditions, and use of air-water temperature relationships derived from daily data developed over the course of three summer months as a surrogate for effects of decadal scale air temperature warming trends on JMMST. Uncertainty in the estimates of summer maximum stream temperatures described above and in S1 and S2 Tables would likely be reduced through the use of additional data collected in unsampled geophysical settings and for other years.

Alternate statistical approaches may also reduce uncertainty in the stream temperature predictions shown here. As a result of flow connectivity, stream temperature observations are often spatially dependent. Because new methods are being developed and applied to account for the spatial connectivity among observations in stream networks [45, 46], spatial autocorrelation can now be leveraged to develop more robust statistical relationships for prediction [47, 48]. We did not consider spatial autocorrelation among the observed temperature sites in this study. The new spatial models perform best when observed data are spatially allocated in a manner that facilitates development of autocorrelation functions derived from a broad range of paired distances. Stream temperature monitoring sites used in this study did not capture the range of paired distances among sites for developing appropriate autocorrelation functions. This is an avenue of future work. Future stream temperature monitoring in the SAM region should consider the potential for developing robust autocorrelation functions that can be used for spatial stream network modeling.

### Management implications

These results will aid U.S. Forest Service managers in the classification of watershed condition in response to human-caused stressors, including, for example, warming, acidification, and/or sedimentation [49] and may be useful for regional forest planning. The U.S. Forest Service is currently applying the regional ANC distribution presented here within the logic-based Ecosystem Management Decision-Support (EMDS) system [50] to evaluate acidification impacts and provide guidance to forest management. The EMDS system can be further used to generate new results by making adjustments to input parameters in a user-friendly environment. The system can thus support development of policy recommendations and aid resource managers who are responsible for the protection and restoration of aquatic ecosystems. The EMDS system allows land managers and policy makers to evaluate ecosystem conditions with respect to decision support metrics such as impacts to ecosystem services and logistical considerations for developing strategic priorities for protection and restoration activities at the landscape scale.

The stream temperature results reported here might also be incorporated into EMDS to inform decision making regarding restoration activities associated with acid-sensitive coldwater species. For example, some managers in the region have active programs to reintroduce the southern strain of brook trout in streams where they have been extirpated. Results shown here provide fisheries managers with first approximations of stream thermal conditions that can be used in conjunction with acidification status to identify appropriate locations for both short-term and long-term success of reintroduction of brook trout or other species. Nevertheless, it will be important to field-verify stream temperature predictions with additional stream water sampling before adjustments to current land management practices are made. Assessments of other aspects of stream condition such as physical habitat structure, stream bed sedimentation, and barriers to movement can also be incorporated into the EMDS system to develop a more comprehensive approach towards aquatic habitat management.

## Conclusions

Our results indicate that climate-induced stream warming and headwater stream acidity represent a significant dual challenge to maintaining suitable habitat for coldwater species in the SAM. This research further suggests that species’ distributional shifts to colder, higher-elevation habitats in response to stream warming can be constrained by acidification of headwater streams. The potential extent of habitat loss from the collective influence of stream warming and stream acidity warrants additional effort to reduce uncertainty in the prediction of spatial patterns of stream temperature and ANC. Results of our analysis identify areas of the SAM where consideration of both stream temperature and stream acidification will be important in developing climate change adaptation plans. To our knowledge, these results represent the first regional assessment of aquatic habitat suitability with respect to the combined effects of stream temperature and acid-base status in the United States. Spatially explicit results will be useful to natural resource management agencies and aquatic biologists for restoration planning, which may include fish stocking, liming to reduce stream water acidity, and riparian canopy enhancement to limit stream warming due to insolation.

## Supporting Information

S1 Fig Predicted vs. observed ANC based on model results for a) the earlier ANC estimates from Povak et al. [23] and b) bias-adjusted ANC estimates developed for this study. (DOCX)

S2 Fig Locations of streams having predicted ANC < 50 μeq/L (red), temperature > 20°C (orange), ANC < 50 μeq/L and temperature > 20°C (pink), and suitable habitat with respect to ANC and temperature (blue). Habitat suitability results are shown for the a) George Washington, b) Jefferson, c) Cherokee, d) Pisgah, e) Nantahala, f) Chattahoochee, and g) Sumter National Forests.
(DOCX)

S1 Table Length of stream and percentage of total stream length that was predicted to be: a) too warm (July mean daily maximum stream water temperature, T, > 18°C), b) too acidic (ANC < 50 μeq/L), or c) suitable (ANC > 50 μeq/L and T < 18°C). (DOCX)

S2 Table Length of stream and percentage of total stream length that was predicted to be: a) too warm (July mean daily maximum stream water temperature, T, > 22°C), b) too acidic (ANC < 50 μeq/L), or c) suitable (ANC > 50 μeq/L and T < 22°C). (DOCX)
