# Supplementary material for: Downstream Warming and Headwater Acidity May Diminish Coldwater Habitat in Southern Appalachian Mountain Streams
Source: PLoS One. 2015 Aug 6;10(8):e0134757. doi: 10.1371/journal.pone.0134757 (PMC4527832; doi:10.1371/journal.pone.0134757)

**S1 Fig.** Predicted vs. observed ANC based on model results for a) the earlier ANC estimates from Povak et al.(23) and b) bias-adjusted ANC estimates developed for this study. Bias adjustments were made according to: PredANC_bias adj_ = PredANC + 0.2415*PredANC – 18.771; where PredANC is the original predicted ANC value (µeq/L) from Povak et al. (1) and PredANC_bias adj_ is the bias-adjusted ANC value. This equation was derived from a linear description of the deviation between the 1:1 agreement of predicted versus observed values (solid black line) and the best fit regression of predicted ANC from the original model versus observed ANC (dashed line).


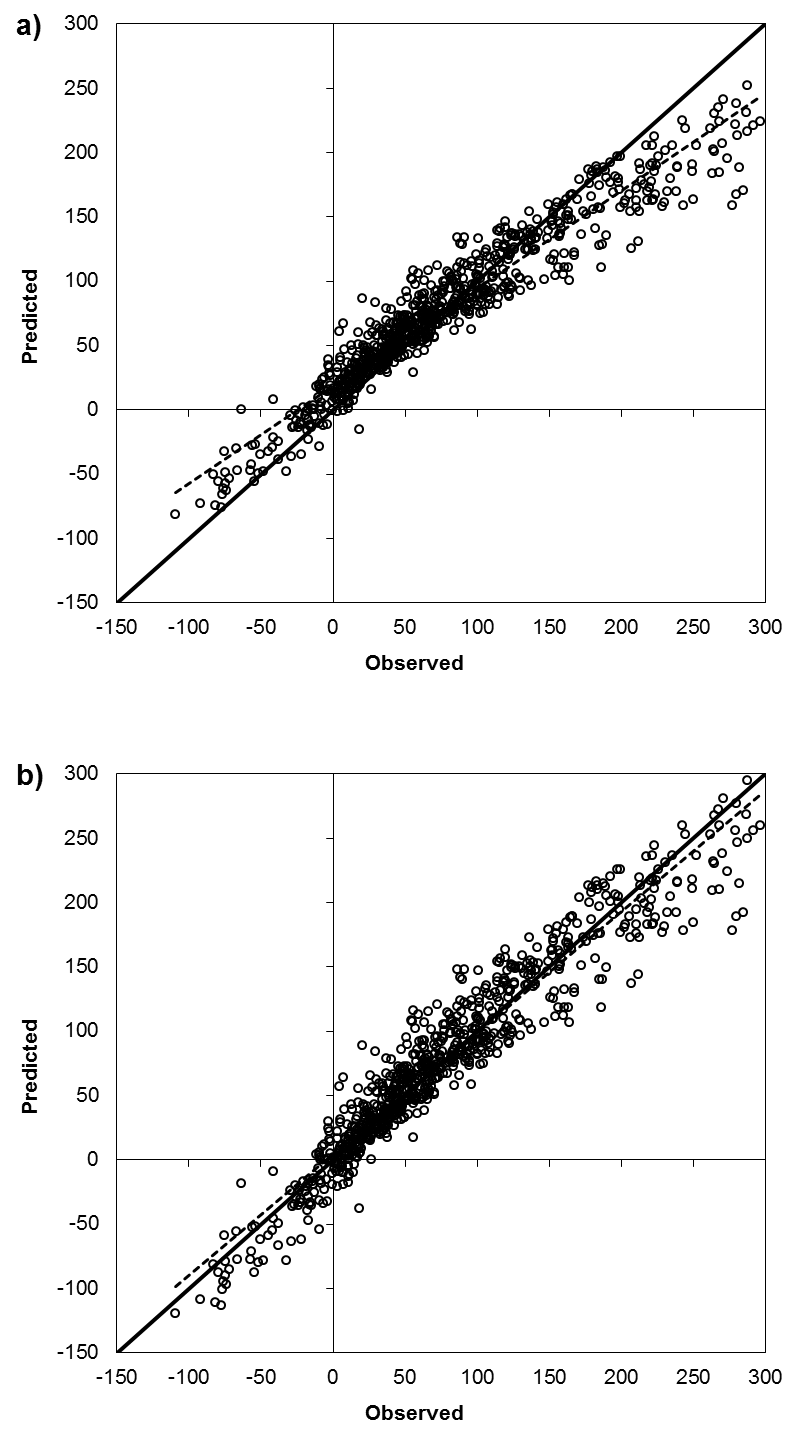

Supplement: S1 Fig — (DOCX) [file pone.0134757.s001.docx]
