# Supplementary material for: Downstream Warming and Headwater Acidity May Diminish Coldwater Habitat in Southern Appalachian Mountain Streams
Source: PLoS One. 2015 Aug 6;10(8):e0134757. doi: 10.1371/journal.pone.0134757 (PMC4527832; doi:10.1371/journal.pone.0134757)

**S2 Fig.** Locations of streams having predicted ANC < 50 µeq/L (red), temperature > 20 ºC (orange), ANC < 50 µeq/L and temperature > 20 ºC (pink), and suitable habitat with respect to ANC and temperature (blue) under contemporary July mean maximum daily air temperature. Habitat suitability results are shown for the a) George Washington, b) Jefferson, c) Cherokee, d) Pisgah, e) Nantahala, f) Chattahoochee, and g) Sumter National Forests. Hydrography data used to express analysis results were obtained from the U.S. Geological Survey high resolution National Hydrography Datatset (NHD) dataset. High resolution NHD is primarily derived from 1:24,000 scale hydrography, although some reporting agencies provide data at different resolutions. Depicted stream density can show abrupt differences as a result of these inconsistencies in data resolution.


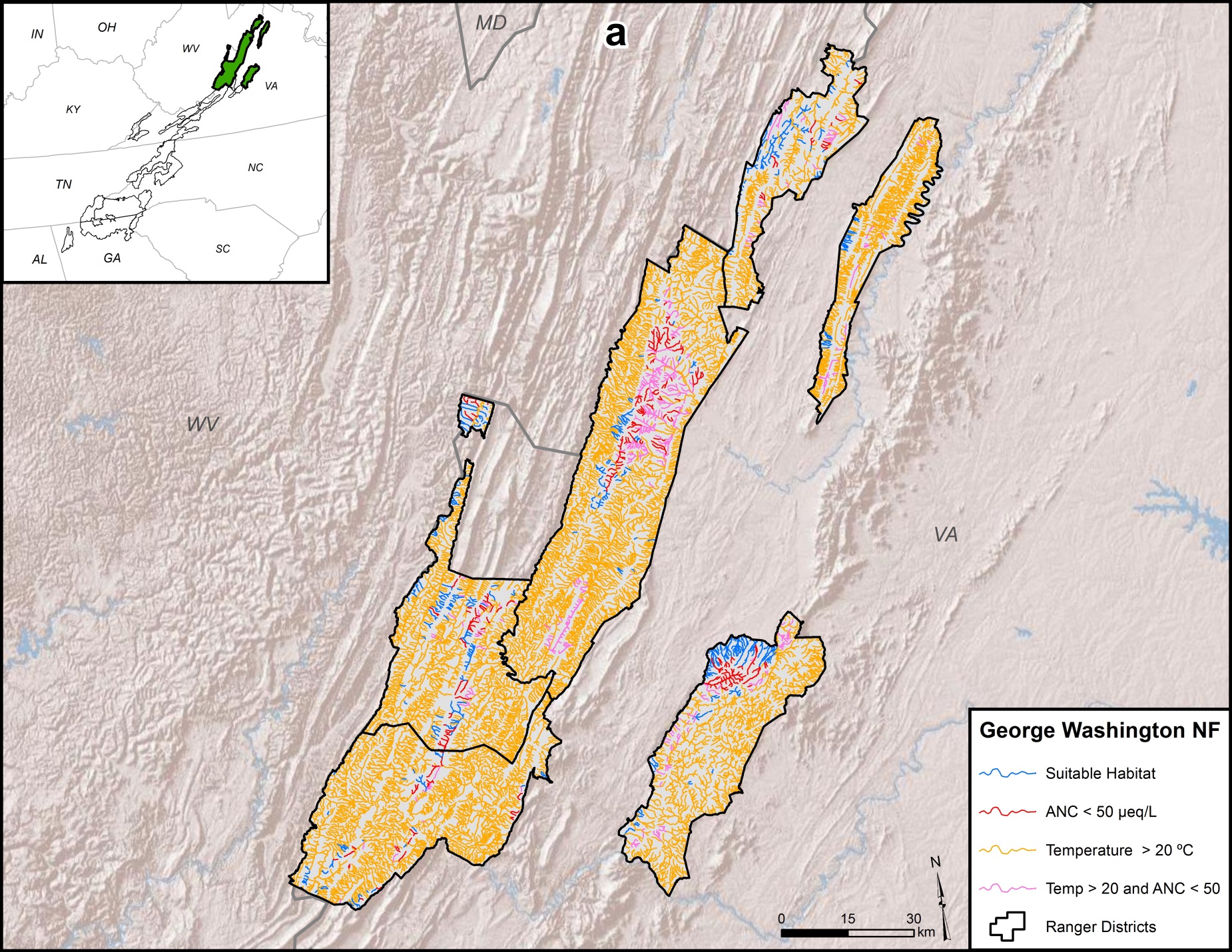


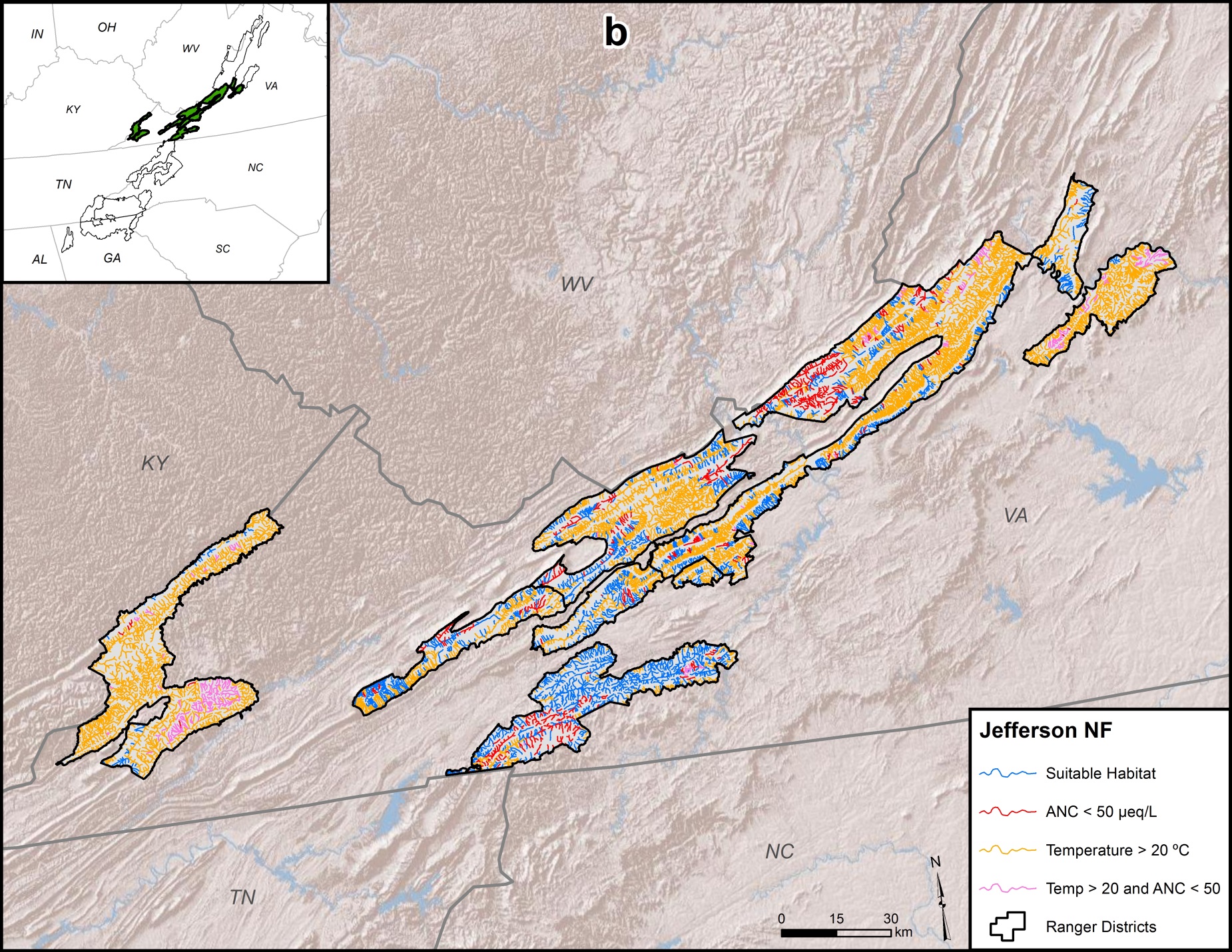


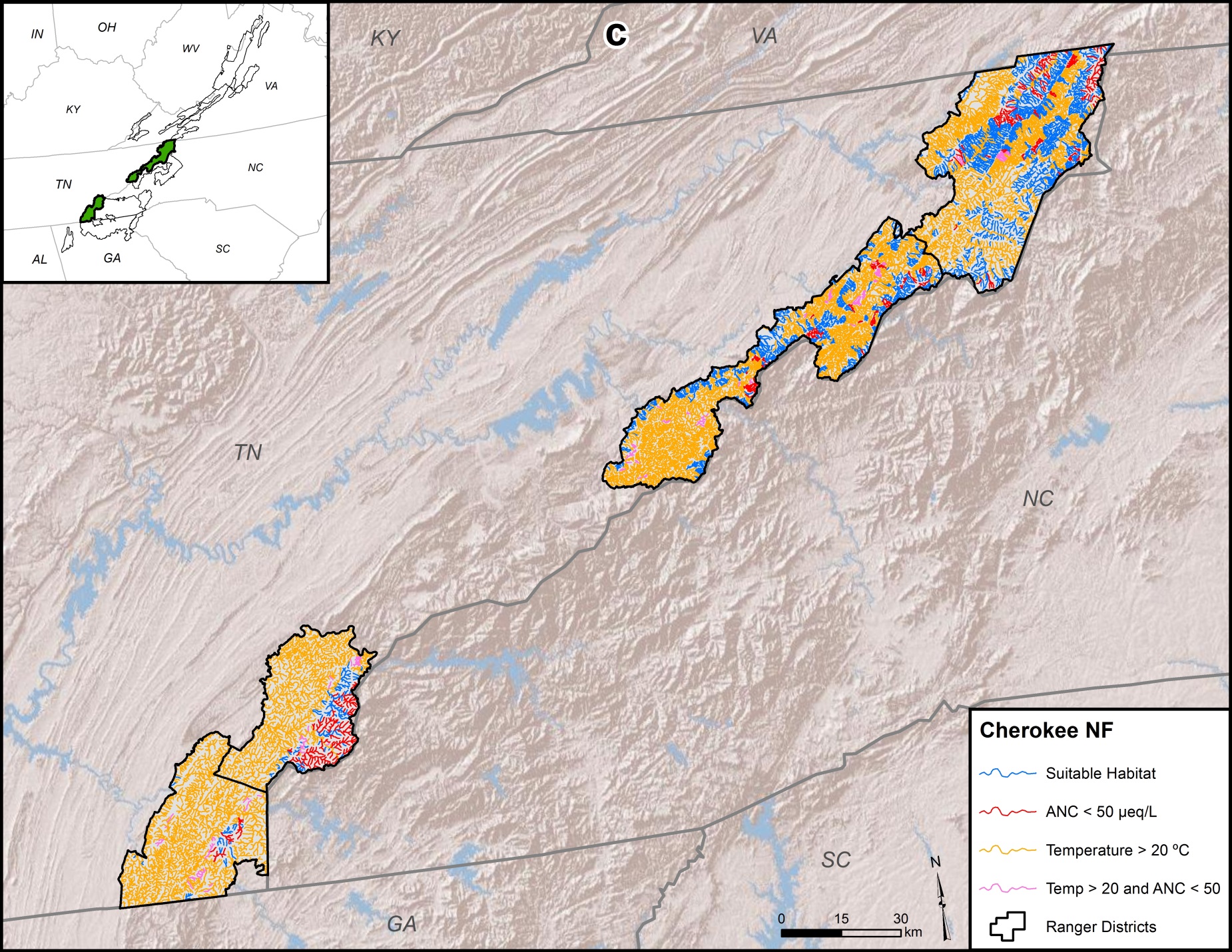

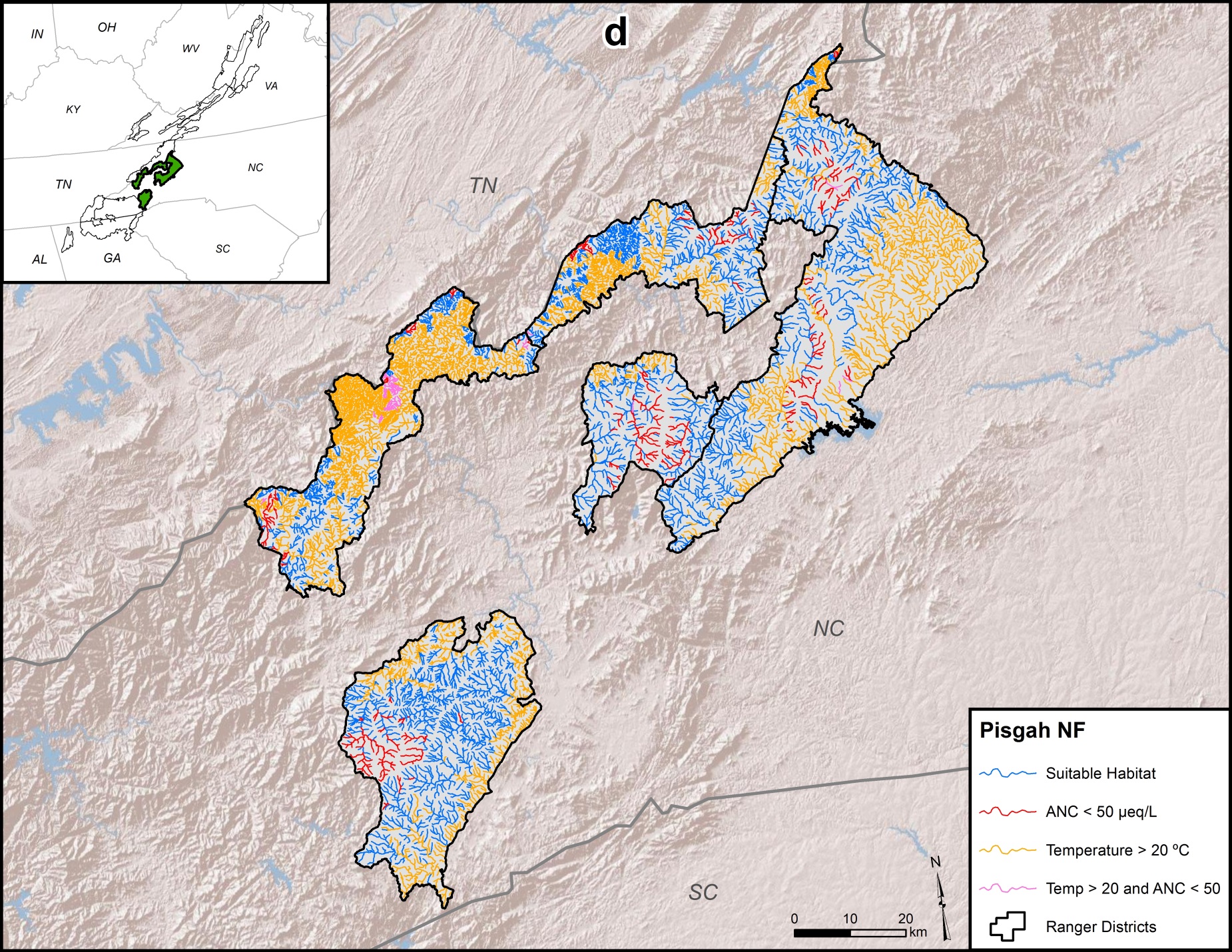


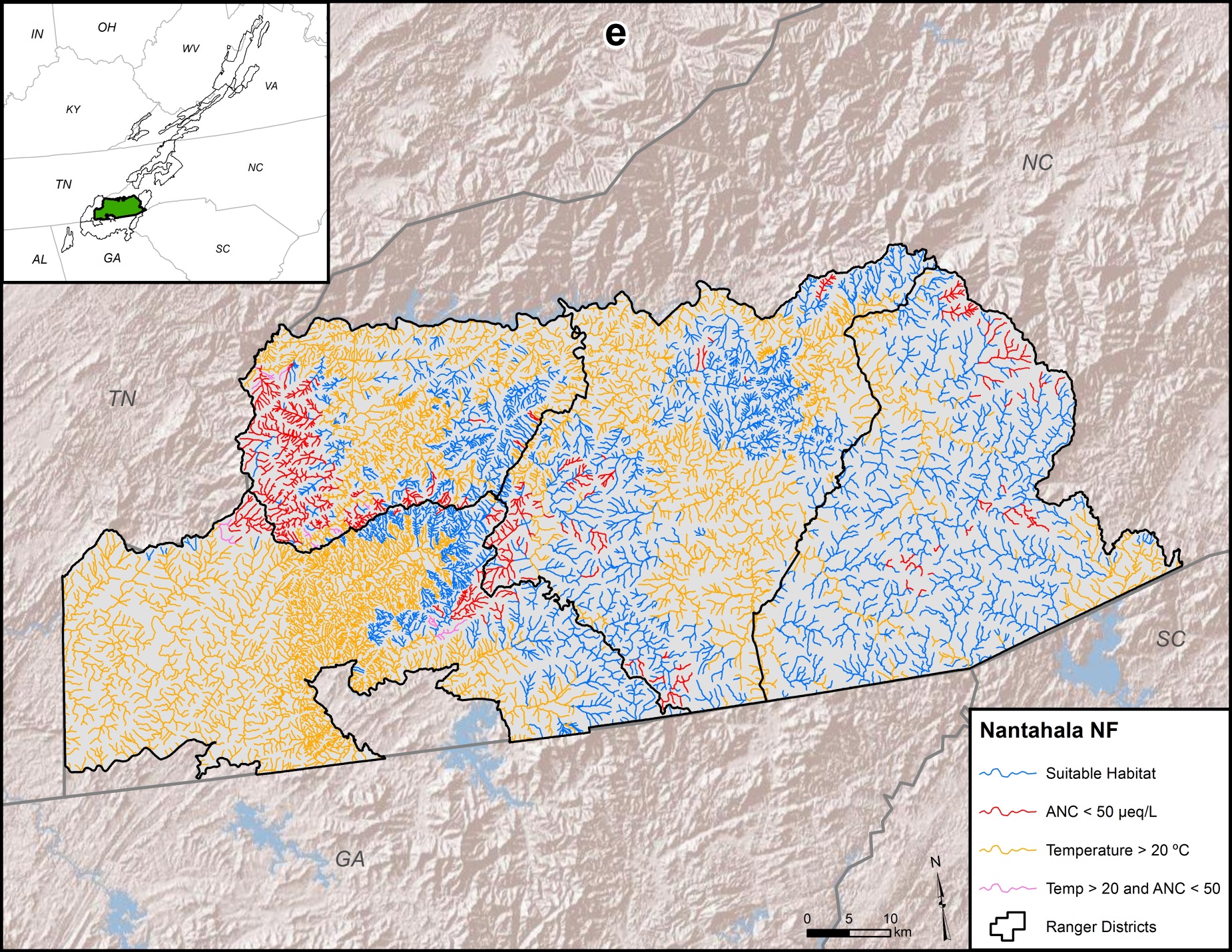


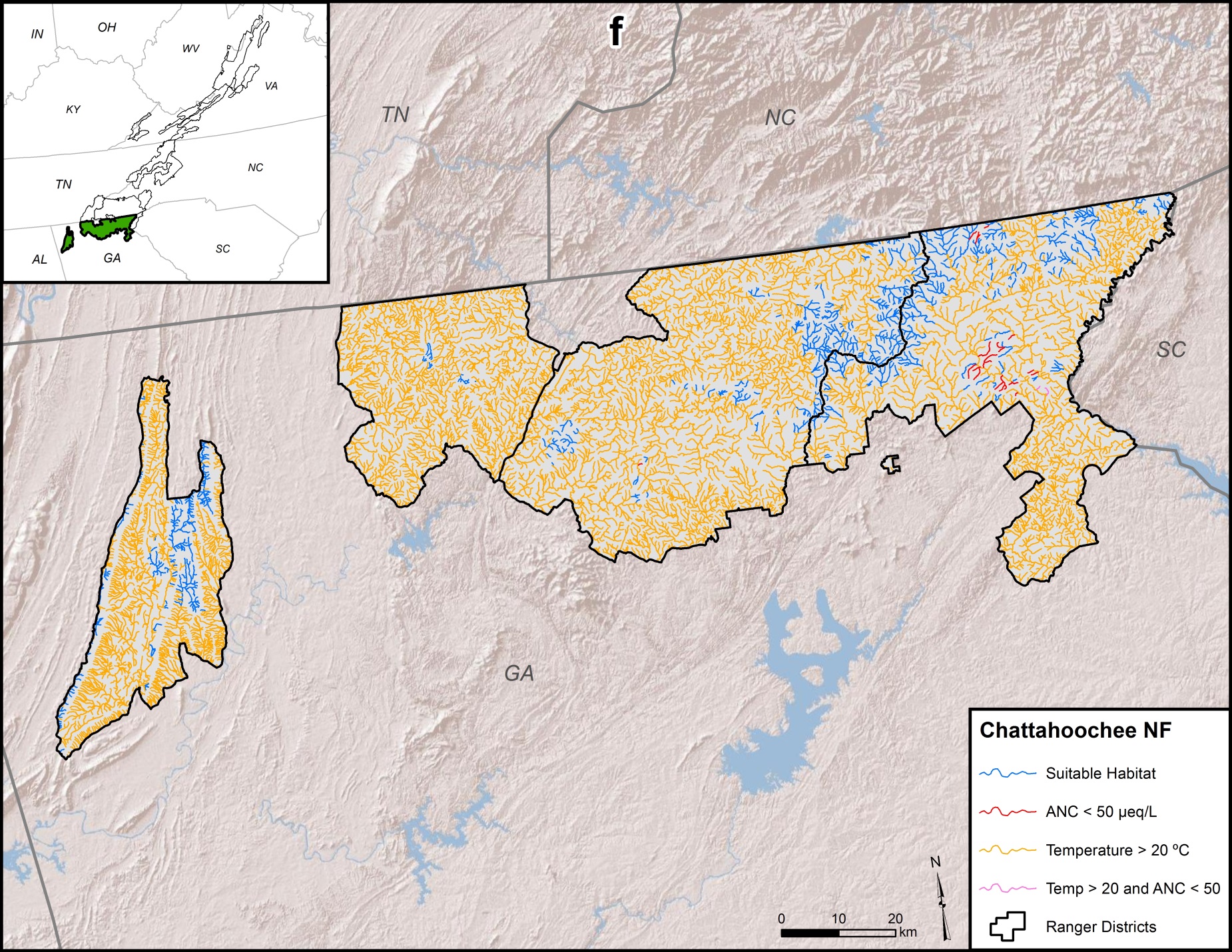


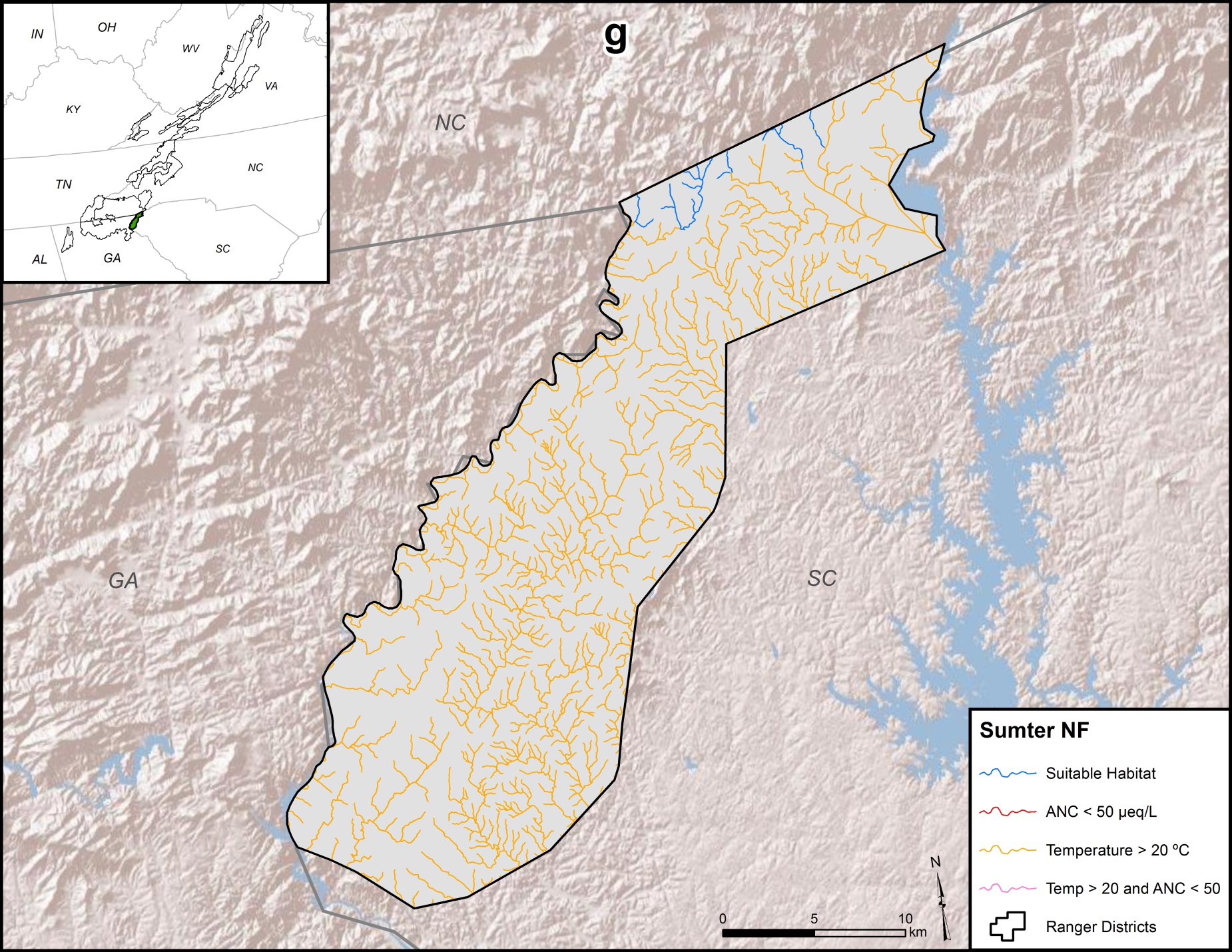

Supplement: S2 Fig — Habitat suitability results are shown for the a) George Washington, b) Jefferson, c) Cherokee, d) Pisgah, e) Nantahala, f) Chattahoochee, and g) Sumter National Forests. (DOCX) [file pone.0134757.s002.docx]
