# Supplementary material for: Downstream Warming and Headwater Acidity May Diminish Coldwater Habitat in Southern Appalachian Mountain Streams
Source: PLoS One. 2015 Aug 6;10(8):e0134757. doi: 10.1371/journal.pone.0134757 (PMC4527832; doi:10.1371/journal.pone.0134757)
Supplement: S1 Table — (DOCX) [file pone.0134757.s003.docx]

| **S1 Table**. Length of stream and percentage of total stream length that was predicted to be: a) too warm (July mean daily maximum stream water temperature (JMMST) > 18 ^o^C), b) too acidic (ANC < 50 µeq/L), or c) suitable (ANC > 50 µeq/L and JMMSTT < 18 ^o^C). | | | | | | | | | | | | | | |
| --- | --- | --- | --- | --- | --- | --- | --- | --- | --- | --- | --- | --- | --- | --- |
| National Forest/  Ranger District |  | Total Stream Length |  | Temp > 18 ^o^C | |  | ANC < 50 µeq/L | |  | Temp > 18 ^o^C and  ANC < 50 µeq/L | |  | Suitable Habitat | |
|  |  | km |  | km | % |  | km | % |  | km | % |  | km | % |
| **George Washington** |  |  |  |  |  |  |  |  |  |  |  |  |  |  |
| James River |  | 2,505 |  | 2,504 | 100.0 |  | 59 | 2.4 |  | 59 | 2.4 |  | 1 | 0.0 |
| Lee |  | 1,904 |  | 1,863 | 97.9 |  | 114 | 6.0 |  | 114 | 6.0 |  | 41 | 2.1 |
| North River |  | 3,857 |  | 3,852 | 99.9 |  | 475 | 12.3 |  | 469 | 12.2 |  | 0 | 0.0 |
| Pedlar |  | 1,678 |  | 1,595 | 95.1 |  | 174 | 10.4 |  | 174 | 10.4 |  | 83 | 4.9 |
| Warm Springs |  | 2,147 |  | 2,140 | 99.7 |  | 118 | 5.5 |  | 111 | 5.2 |  | 0 | 0.0 |
| TOTAL |  | **12,090** |  | **11,954** | 98.9 |  | **939** | 7.8 |  | **927** | 7.7 |  | **124** | 1.0 |
| **Jefferson** |  |  |  |  |  |  |  |  |  |  |  |  |  |  |
| Clinch |  | 1,851 |  | 1,824 | 98.6 |  | 219 | 11.8 |  | 219 | 11.8 |  | 27 | 1.4 |
| Eastern Divide |  | 5,236 |  | 5,061 | 96.7 |  | 559 | 10.7 |  | 466 | 8.9 |  | 82 | 1.6 |
| Glenwood |  | 1,139 |  | 1,100 | 96.6 |  | 97 | 8.5 |  | 97 | 8.5 |  | 39 | 3.4 |
| Mt. Rogers |  | 2,630 |  | 2,001 | 76.1 |  | 293 | 11.1 |  | 172 | 6.5 |  | 508 | 19.3 |
| TOTAL |  | **10,856** |  | **9,986** | 92.0 |  | **1,168** | 10.8 |  | **954** | 8.8 |  | **656** | 6.0 |
| **Cherokee** |  |  |  |  |  |  |  |  |  |  |  |  |  |  |
| Nolichucky |  | 3,927 |  | 3,818 | 97.2 |  | 222 | 5.6 |  | 201 | 5.1 |  | 88 | 2.2 |
| Ocoee |  | 1,696 |  | 1,696 | 100.0 |  | 76 | 4.5 |  | 76 | 4.5 |  | 0 | 0.0 |
| Tellico |  | 1,507 |  | 1,463 | 97.1 |  | 217 | 14.4 |  | 182 | 12.1 |  | 8 | 0.5 |
| Watauga |  | 4,053 |  | 3,696 | 91.2 |  | 284 | 7.0 |  | 236 | 5.8 |  | 310 | 7.6 |
| TOTAL |  | **11,183** |  | **10,674** | 95.4 |  | **798** | 7.1 |  | **694** | 6.2 |  | **406** | 3.6 |
| **Pisgah** |  |  |  |  |  |  |  |  |  |  |  |  |  |  |
| Appalachian |  | 3,968 |  | 3,400 | 85.7 |  | 315 | 7.9 |  | 126 | 3.2 |  | 379 | 9.5 |
| Grandfather |  | 2,330 |  | 2,127 | 91.3 |  | 138 | 5.9 |  | 88 | 3.8 |  | 153 | 6.6 |
| Pisgah |  | 1,706 |  | 1,286 | 75.4 |  | 115 | 6.7 |  | 8 | 0.5 |  | 313 | 18.4 |
| TOTAL |  | **8,004** |  | **6,813** | 85.1 |  | **568** | 7.1 |  | **222** | 2.8 |  | **845** | 10.6 |
| **Nantahala** |  |  |  |  |  |  |  |  |  |  |  |  |  |  |
| Cheoah |  | 1,768 |  | 1,722 | 97.4 |  | 355 | 20.1 |  | 340 | 19.3 |  | 31 | 1.8 |
| Nantahala-Highlands |  | 1,638 |  | 1,168 | 71.3 |  | 111 | 6.8 |  | 9 | 0.5 |  | 367 | 22.4 |
| Nantahala-Wayah |  | 2,573 |  | 2,185 | 84.9 |  | 156 | 6.1 |  | 81 | 3.2 |  | 314 | 12.2 |
| Tusquitee |  | 2,999 |  | 2,952 | 98.4 |  | 105 | 3.5 |  | 100 | 3.3 |  | 42 | 1.4 |
| TOTAL |  | **8,978** |  | **8,026** | 89.4 |  | **728** | 8.1 |  | **531** | 5.9 |  | **754** | 8.4 |
| **Chattahoochee** |  |  |  |  |  |  |  |  |  |  |  |  |  |  |
| Blue Ridge |  | 3,285 |  | 3,277 | 99.8 |  | 1 | 0.0 |  | 1 | 0.0 |  | 8 | 0.2 |
| Chattooga River |  | 2,371 |  | 2,346 | 98.9 |  | 42 | 1.8 |  | 36 | 1.5 |  | 19 | 0.8 |
| Conasauga |  | 3,362 |  | 3,313 | 98.6 |  | 0 | 0.0 |  | 0 | 0.0 |  | 48 | 1.4 |
| TOTAL |  | **9,017** |  | **8,936** | 99.1 |  | **42** | 0.5 |  | **36** | 0.4 |  | **75** | 0.8 |
| **Sumter** |  |  |  |  |  |  |  |  |  |  |  |  |  |  |
| Andrew Pickens |  | 906 |  | 906 | 100.0 |  | 0 | 0.0 |  | 0 | 0.0 |  | 0 | 0.0 |
| TOTAL |  | **906** |  | **906** | 100.0 |  | **0** | 0.0 |  | **0** | 0.0 |  | **0** | 0.0 |
