# Supplementary material for: Downstream Warming and Headwater Acidity May Diminish Coldwater Habitat in Southern Appalachian Mountain Streams
Source: PLoS One. 2015 Aug 6;10(8):e0134757. doi: 10.1371/journal.pone.0134757 (PMC4527832; doi:10.1371/journal.pone.0134757)
Supplement: S2 Table — (DOCX) [file pone.0134757.s004.docx]

| **S2 Table.** Length of stream and percentage of total stream length that was predicted to be: a) too warm (July mean daily maximum stream temperature (JMMST) > 22 ^o^C), b) too acidic (ANC < 50 µeq/L), or c) suitable (ANC > 50 µeq/L and JMMST < 22 ^o^C). | | | | | | | | | | | | | | |
| --- | --- | --- | --- | --- | --- | --- | --- | --- | --- | --- | --- | --- | --- | --- |
| National Forest/  Ranger District |  | Total Stream Length |  | Temp > 22 ^o^C | |  | ANC < 50 µeq/L | |  | Temp > 22 ^o^C and  ANC < 50 µeq/L | |  | Suitable Habitat | |
|  |  | km |  | km | % |  | km | % |  | km | % |  | km | % |
| **George Washington** |  |  |  |  |  |  |  |  |  |  |  |  |  |  |
| James River |  | 2,505 |  | 1,652 | 65.9 |  | 59 | 2.4 |  | 0 | 0.0 |  | 794 | 31.7 |
| Lee |  | 1,904 |  | 931 | 48.9 |  | 114 | 6.0 |  | 0 | 0.0 |  | 859 | 45.1 |
| North River |  | 3,857 |  | 2,017 | 52.3 |  | 475 | 12.3 |  | 1 | 0.0 |  | 1,367 | 35.4 |
| Pedlar |  | 1,678 |  | 706 | 42.1 |  | 174 | 10.4 |  | 11 | 0.6 |  | 808 | 48.2 |
| Warm Springs |  | 2,147 |  | 1,093 | 50.9 |  | 118 | 5.5 |  | 2 | 0.1 |  | 938 | 43.7 |
| TOTAL |  | **12,090** |  | **6,399** | 52.9 |  | **939** | 7.8 |  | **14** | 0.1 |  | **4,766** | 39.4 |
| **Jefferson** |  |  |  |  |  |  |  |  |  |  |  |  |  |  |
| Clinch |  | 1,851 |  | 1,194 | 64.5 |  | 219 | 11.8 |  | 25 | 1.4 |  | 463 | 25.0 |
| Eastern Divide |  | 5,236 |  | 1,614 | 30.8 |  | 559 | 10.7 |  | 1 | 0.0 |  | 3,064 | 58.5 |
| Glenwood |  | 1,139 |  | 496 | 43.5 |  | 97 | 8.5 |  | 2 | 0.2 |  | 549 | 48.2 |
| Mt. Rogers |  | 2,630 |  | 205 | 7.8 |  | 293 | 11.1 |  | 0 | 0.0 |  | 2,132 | 81.1 |
| TOTAL |  | **10,856** |  | **3,508** | 32.3 |  | **1,168** | 10.8 |  | **28** | 0.3 |  | **6,208** | 57.2 |
| **Cherokee** |  |  |  |  |  |  |  |  |  |  |  |  |  |  |
| Nolichucky |  | 3,927 |  | 1,071 | 27.3 |  | 222 | 5.6 |  | 3 | 0.1 |  | 2,636 | 67.1 |
| Ocoee |  | 1,696 |  | 1,203 | 70.9 |  | 76 | 4.5 |  | 0 | 0.0 |  | 418 | 24.6 |
| Tellico |  | 1,507 |  | 886 | 58.8 |  | 217 | 14.4 |  | 0 | 0.0 |  | 404 | 26.8 |
| Watauga |  | 4,053 |  | 924 | 22.8 |  | 284 | 7.0 |  | 0 | 0.0 |  | 2,845 | 70.2 |
| TOTAL |  | **11,183** |  | **4,084** | 36.5 |  | **798** | 7.1 |  | **3** | 0.0 |  | **6,304** | 56.4 |
| **Pisgah** |  |  |  |  |  |  |  |  |  |  |  |  |  |  |
| Appalachian |  | 3,968 |  | 773 | 19.5 |  | 315 | 7.9 |  | 2 | 0.0 |  | 2,882 | 72.6 |
| Grandfather |  | 2,330 |  | 437 | 18.7 |  | 138 | 5.9 |  | 0 | 0.0 |  | 1,755 | 75.3 |
| Pisgah |  | 1,706 |  | 144 | 8.4 |  | 115 | 6.7 |  | 0 | 0.0 |  | 1,447 | 84.8 |
| TOTAL |  | **8,004** |  | **1,353** | 16.9 |  | **568** | 7.1 |  | **2** | 0.0 |  | **6,085** | 76.0 |
| **Nantahala** |  |  |  |  |  |  |  |  |  |  |  |  |  |  |
| Cheoah |  | 1,768 |  | 269 | 15.2 |  | 355 | 20.1 |  | 0 | 0.0 |  | 1,144 | 64.7 |
| Nantahala-Highlands |  | 1,638 |  | 113 | 6.9 |  | 111 | 6.8 |  | 0 | 0.0 |  | 1,414 | 86.3 |
| Nantahala-Wayah |  | 2,573 |  | 603 | 23.4 |  | 156 | 6.1 |  | 0 | 0.0 |  | 1,815 | 70.5 |
| Tusquitee |  | 2,999 |  | 1,387 | 46.2 |  | 105 | 3.5 |  | 0 | 0.0 |  | 1,507 | 50.2 |
| TOTAL |  | **8,978** |  | **2,371** | 26.4 |  | **728** | 8.1 |  | **0** | 0.0 |  | **5,879** | 65.5 |
| **Chattahoochee** |  |  |  |  |  |  |  |  |  |  |  |  |  |  |
| Blue Ridge |  | 3,285 |  | 1,467 | 44.7 |  | 1 | 0.0 |  | 0 | 0.0 |  | 1,817 | 55.3 |
| Chattooga River |  | 2,371 |  | 1,101 | 46.4 |  | 42 | 1.8 |  | 0 | 0.0 |  | 1,228 | 51.8 |
| Conasauga |  | 3,362 |  | 2,331 | 69.3 |  | 0 | 0.0 |  | 0 | 0.0 |  | 1,031 | 30.7 |
| TOTAL |  | **9,017** |  | **4,899** | 54.3 |  | **42** | 0.5 |  | **0** | 0.0 |  | **4,076** | 45.2 |
| **Sumter** |  |  |  |  |  |  |  |  |  |  |  |  |  |  |
| Andrew Pickens |  | 906 |  | 605 | 66.8 |  | 0 | 0.0 |  | 0 | 0.0 |  | 301 | 33.2 |
| TOTAL |  | **906** |  | **605** | 66.8 |  | **0** | 0.0 |  | **0** | 0.0 |  | **301** | 33.2 |
